# Virtual Screening of Peptide Libraries: The Search for Peptide-Based Therapeutics Using Computational Tools

**DOI:** 10.3390/ijms25031798

**Published:** 2024-02-01

**Authors:** Marian Vincenzi, Flavia Anna Mercurio, Marilisa Leone

**Affiliations:** Institute of Biostructures and Bioimaging, Via Pietro Castellino 111, 80131 Naples, Italy; marian.vincenzi@ibb.cnr.it (M.V.); flaviaanna.mercurio@cnr.it (F.A.M.)

**Keywords:** bioactive peptides, PPIs, drug discovery, virtual screening, anticancer peptides, antiviral peptides

## Abstract

Over the last few decades, we have witnessed growing interest from both academic and industrial laboratories in peptides as possible therapeutics. Bioactive peptides have a high potential to treat various diseases with specificity and biological safety. Compared to small molecules, peptides represent better candidates as inhibitors (or general modulators) of key protein–protein interactions. In fact, undruggable proteins containing large and smooth surfaces can be more easily targeted with the conformational plasticity of peptides. The discovery of bioactive peptides, working against disease-relevant protein targets, generally requires the high-throughput screening of large libraries, and in silico approaches are highly exploited for their low-cost incidence and efficiency. The present review reports on the potential challenges linked to the employment of peptides as therapeutics and describes computational approaches, mainly structure-based virtual screening (SBVS), to support the identification of novel peptides for therapeutic implementations. Cutting-edge SBVS strategies are reviewed along with examples of applications focused on diverse classes of bioactive peptides (i.e., anticancer, antimicrobial/antiviral peptides, peptides blocking amyloid fiber formation).

## 1. Introduction

Protein functions are primarily dictated by interactions with other proteins or biomolecules [1]. Interestingly, an ensemble of hundreds of thousands of protein–protein interactions (PPIs) composes the human interactome, and the aberrant modulation, in particular inhibition, of key PPIs within this ensemble can be associated with different diseases, ranging from cancer to neurodegenerative pathologies [2,3]. Since large regions mediate PPIs, and they are usually not provided with well-defined pockets, grooves or clefts, their targeting by small molecules represents a very challenging drug development route [2,3]. Both natural and synthetic peptides represent valuable alternatives to small molecules in the development of therapeutic agents able to inhibit PPIs [2]. In fact, due to the different chemical–physical properties with respect to small molecules, peptides can better adapt to the large interaction surfaces of proteins [2,3,4]. In addition, the possibility of improved peptide ADME (i.e., absorption, distribution, metabolism, excretion) profiles represents a further justification for the interest of drug development research in peptides targeting PPIs [2,3]. Peptides with a small molecular weight, high flexibility, and minimal toxicity can potentially constitute a new class of biopharmaceuticals [3]. On the other hand, peptide-based drug development, which requires analysis by high-throughput (HT) approaches of peptide–protein interactions, represents an expensive and time-consuming process that can benefit from in silico methods [1]. Therefore, this review is focused on the cutting-edge in silico approaches that could be implemented to discover peptide modulators of PPIs with potential therapeutic effects. The applications of computational methods centered around diverse classes of peptides (including anticancer, antiviral, self-assembling peptides) are also discussed.

### 1.1. Targeting PPIs with Peptides

The availability of 3D protein structures, principally protein–protein complexes, is crucial to developing peptides targeting PPIs [1,2]. In this framework, Nuclear Magnetic Resonance (NMR), X-ray crystallography, and cryo-Electron Microscopy (cryo-EM) are the most frequently applied techniques to obtain the structures of single interactors or their complexes [1,2]. More precisely, X-ray crystallography is rather suitable for retrieving data for large globular domains (alone or associated with each other) whereas, NMR can also be exploited to study proteins involved in the formation of transient and weak complexes and obtain dynamic information [1,2]. Moreover, structures with near-atomic resolutions can be determined by combining low-resolution structural techniques [e.g., cryo-Electron Microscopy (cryo-EM) and Small-Angle X-ray Scattering (SAXS)] with X-ray crystallography, NMR, Förster Resonance Energy Transfer (FRET), or Mass Spectroscopy (MS) techniques, along with in silico approaches [2]. The reference database for 3D structures obtained by all these techniques is the Protein Data Bank (PDB) [3]. With a 3D structure in hand, a detailed analysis of the structural features characterizing the protein interfaces can be carried out, and the gained structural insights can be employed for the design of peptides able to interfere with the PPI under analysis. In the peptide drug design field, in silico approaches to predicting protein–peptide complexes offer some advantages if compared to experimental methods, although they still present diverse challenges to overcome [1]. A significant issue, which should be taken into account when trying to computationally model protein–peptide interactions, relies on the variety of conformations that can be explored by both proteins and peptides following mutual binding (the so-called induced fit) and the difficulties in properly ranking the in silico predicted solutions for a specific protein–peptide complex [1]. In general, it is not easy to guess in silico which protein fragment will bind a protein partner and establish which conformation the selected peptide region will exactly assume upon complex formation [1].

However, the combination of in silico and experimental methodologies is the most common strategy in drug discovery as it can exploit an initial generation of hypotheses by computational tools, thus greatly speeding up the overall peptide identification process and reducing monetary costs. In silico strategies work better when at least the 3D structures of isolated target proteins are available and have been experimentally determined, although, recently, the AlphaFold2 approach has tremendously revolutionized the world of molecular modeling by improving prediction of 3D structures [5,6,7]. Nevertheless, recent advances have been made for the de novo design of new proteins through deep learning approaches, including denoising diffusion probabilistic models (DDPMs) [8]. For example, the recently described “RoseTTAFold diffusion” (RFdiffusion) relies on a generative model of protein backbones. It can be very powerful for the design of protein monomers, protein interactors, oligomers with precise symmetries, and for scaffolding enzyme active pockets and symmetric motifs when trying to build therapeutic proteins or those able to bind metals [8].

When the identification of bioactive peptides targeting certain PPIs is negatively affected by the unavailability of 3D structures, phenotype- and target-oriented approaches often constitute valuable solutions [2]. The phenotype-oriented approach [9] may include screening natural peptides or synthetic libraries, the members of which are synthesized on a solid support. This approach offers the chance to simultaneously evaluate a considerable number of peptide variants. The phage display technology, that relies on screening peptides located on the surface of filamentous bacteriophages expressing them, represents another alternative to identify bioactive peptides [2,4,10]. Regarding target-oriented approaches, PEPscan is a valuable method to identify peptide ligands by scanning consecutive and overlapped peptide fragments that derive from one of the two proteins participating in the PPI of interest [2]. PEPscan relies on peptide arrays that can be obtained through the SPOT technique and offers access to a wide variety of peptide sizes (generally, lengths range from 5 to 30 amino acids) and formats [2,11].

The pepATTRACT webserver can be a valuable in silico tool that resembles the experimental PEPscan approach. However, pepATTRACT requires knowledge of the 3D structure of the target protein to be used, along with peptide sequences, as input to perform blind docking runs [12].

Another crucial point when searching for novel peptide modulators of PPIs is the biophysical characterization of peptide–protein and protein–protein interactions, including the determination of binding affinities and stoichiometries. To this aim, many biophysical techniques can be implemented, but, among the most widely used, there are Isothermal Titration Calorimetry (ITC) [13], Surface Plasmon resonance (SPR) [14], BioLayer Interferometry (BLI) [15], and MicroScale Thermophoresis (MST) [16]. For weak binding and under precise conditions, NMR techniques can be supportive to analyze protein–ligand complexes better and can even be used to estimate the dissociation constants [17,18,19,20,21].

ITC is very useful for determining binding stoichiometry, equilibrium constants, and the variation in enthalpy (ΔH) and entropy (ΔS) associated with molecular recognition, whereas the rates of association (*k*_on_) and dissociation (*k*_off_) characterizing PPIs can be determined by SPR [2].

Once a peptide modulator of a certain PPI is identified, it is important to chemically optimize it. The first crucial step consists of the improvement of the interaction affinity. The peptide segments extracted from the whole protein structure organization tend to be disordered in solution but might assume a more ordered bioactive conformation when in complex with the interaction partner [22]. Thus, forming a protein–peptide complex can often be associated with a high entropic cost due to the absence of a stable conformation in the peptide-unbound state and the rise of a more constrained structure in the peptide-bound form. Therefore, strategies for optimizing peptides targeting PPIs should include those modifications that can induce lower entropy costs and/or higher enthalpic energies of binding. To reach such a goal, it can be useful to constrain the peptide architecture and eventually favor the rise in the unbound form of certain secondary structure elements, for example, through cyclization, which could be achieved by the formation of amide bonds, disulfide bridges, or the generation of stapled peptides [2,23,24,25,26]. Nevertheless, as introduced before, one of the difficulties of targeting PPIs is the extension of the interacting surfaces, and macrocyclization is described as a promising solution to make peptides better suited to adapt to large surfaces and consequently work efficiently as PPI inhibitors [24]. Nevertheless, chemical modifications can also be employed to improve the pharmacological properties of model peptides [2]. In fact, during peptide optimization, particular attention must be given to peptide cell permeability and resistance to protease degradation, and many medicinal chemistry approaches have already been proposed to improve such properties (see below) [27,28,29,30,31,32,33].

The following section will describe the different features characterizing peptides as therapeutics and strategies to improve peptide drug-like characteristics.

### 1.2. Brief Overview of Peptide Therapeutic Potential: Major Drawbacks and Optimization Routes

The use of peptides to develop original therapeutics is supported by their potentially desirable features, including high binding affinities and large selectivity, that can be connected to the relatively big sizes with respect to small compounds and, consequently, the capacity to establish an increased number of intermolecular interactions with the target proteins [34,35].

The hormone insulin is a peptide made up of 51 amino acids. The extraction of insulin from the pancreas of animals and its first employment in the treatment of diabetes back in 1920 represents a milestone in the field of peptide-based therapeutics [34,35,36]. Another example of a therapeutic peptide isolated from natural sources is the adrenocorticotrophic hormone (ACTH) that is effective in treating different pathologies of the endocrine system [34]. Interestingly, bioactive peptides have also been identified in exotic reservoirs, such as venoms from arthropods and cephalopods [34].

Over the years, tremendous improvements have been achieved concerning peptide synthesis methodologies and automation, favoring research in the field of peptide therapeutics. Peptides started to attract attention in 1963 after Merrifield introduced solid-phase peptide synthesis (SPPS) [33,35,36]. SPPS laid the foundation for the development of an automatic peptide synthesizer by joining both amino acid coupling and deprotection in a single reactor, thus enormously simplifying the production of diverse peptide sequences [33]. Later in the 1980s, the generation of larger pure peptides became possible thanks to the emergence of recombinant technology [36]. Another leap forward in the field of therapeutic peptides was reached with the development of strategies to increase molecular weight by the conjugation of peptides to lipids, bigger proteins, and polyethylene glycol. Such conjugated systems paved the way to enhanced renal clearance and plasma circulation times with respect to unmodified peptides that are instead quickly (i.e., within minutes) cleared from plasma [36].

Later, the progress of combinatorial synthetic libraries and the introduction of in vitro display technologies (e.g., phage display, yeast display, mRNA display, ribosome display, and DNA display) drove the identification of original peptides for a larger set of targets [35]. Intriguingly, display techniques create a sort of bridge between the phenotype (i.e., peptide) and genotype (RNA/DNA coding sequence), thus allowing peptide selection based on affinity while genetic material can be recovered and amplified through PCR (Polymerase Chain Reaction) or upon infection of host cells by phage virions [36].

As regards peptides from the natural reservoir (also named “native” peptides), a major issue that can be encountered in employing them as therapeutic agents is the disadvantageous absorption, distribution, metabolism, and excretion (ADME) profile [34,35]. Indeed, peptides generally do not respect the so-called rule of 5 established for small molecules by Lipinski (i.e., molecular weight < 500 Da, number of H-bond donors ≤ 5, number of H-bond acceptors ≤ 10, and a partition coefficient logP ≤ 5) and thus are not likely to passively cross membranes and show good oral bioavailability [35]. Diverse studies have demonstrated how the characteristic peptide flexibility could contribute to low passive permeability, thus suggesting the introduction of structural constraints to improve this unfavorable peptide property [35]. Among the solutions proposed to decrease flexibility, the introduction of modified residues (e.g., α-methyl, α,β-dehydro, and β-substituted amino acids) and the substitution of amide bonds with isosteres (e.g., CH_2_O, CH_2_CH_2_, CH_2_NH, CSNH, CH_2_S) represent only a few examples [37]. Cyclization is another strategy proposed to favor a lowering of the peptide flexibility and is therefore indicated as a promising tool to enhance peptide permeability [10,35,37,38].

A further tactic that could increase peptide permeability consists of the N-methylation of backbone nitrogen atoms, which favors an increase in the *cis* peptide bond population by decreasing the energy difference between the cis and trans configurations. Furthermore, this modification also reduces the number of NH backbone groups available to form H-bonds and, consequently, the energy loss related to the desolvation needed for membrane crossover. The capacity of peptides to pass through biological membranes can be improved by conjugation to the so-called cell-penetrating peptides (CPPs), which are generally made up of 5–30 residues and characterized by the presence of basic residues (i.e., arginines and lysines) [35].

Another issue related to peptide therapeutics is the poor resistance to protease digestion. In this context, one of the proposed strategies to increase peptide stability consists of the substitution of L-amino acids, which characterize “native” peptides and can be recognized by proteases with non-natural D-amino acids [10,33,35]. The N-methylation, as well as other N-alkylation modifications, by favoring the *cis* configuration of peptide bonds, can not only improve permeability, as mentioned above, but also stability. The β-amino acids (i.e., β^2^ and β^3^) and γ-amino acids possess additional carbon atoms in the backbone and are proposed as a possible route to make peptides more resistant to protease attack [33,35]. The replacement of peptide bonds with isosteres (e.g., azapeptides and peptoids) represents a promising route to mimic the transition state of peptide bond cleavage, and thus, can be considered a way to inhibit proteases [2,35]. Additional modifications are recognized as stability enhancers and include the capping of the N-terminus, deamination, the extension of the N- and C-extremities, and the insertion of moieties able to simulate disulfide bonds [36]. The peptide’s resistance to protease and its half-life can be improved as well by linkage to elements providing a sort of shielding effect for enzymatic cleavage, such as polyethylene glycol and proteins like the Fc (Fragment crystallizable) domains [35].

Similarly, conjugation to the proopiomelamocortin-derived peptide sequence (i.e., NSSSSGSSGAGQ) and lipids (e.g., C14/16/18 fatty acids) has been proven to be effective in improving the plasma stability [33,34,35,39].

As briefly mentioned before, cyclization is a common strategy to improve peptide stabilities by inducing the formation of secondary structure elements [33,35,38,40]. The stapling of peptides is a widely employed approach that can not only be implemented to enhance peptide resistance to proteases but also cell permeability and target affinity through the introduction of covalent crosslinks that can favor the rise of helical structures [33,36]. Covalent crosslinks can be achieved through a variety of approaches, for example, by the formation of lactam bridges between the side chains of acidic residues (i.e., aspartic acid or glutamic acid) and lysines or by replacing certain residues with cysteines or homocysteines, that can be exploited to obtain disulfide bonds, whereas, several biselectrophilic linkers are available and can be used to introduce a clip by reacting with cysteine residues on a model peptide [33]. In addition, hydrocarbon-stapled peptides can be achieved by an olefinic linkage, obtained through click chemistry, between the side chains of α,α-disubstituted non-natural amino acids, that carry olefinic moieties and are introduced in the i,i+4 or i,i+7 positions of the chosen peptide sequence [41].

The inclusion in a peptide fragment of residues with D-configuration (mainly D-Proline) might favor turn formation and consequently stabilize β-sheet structural organization; the “D-Proline-L-Proline” dipeptide motif can be exploited as well to stabilize β-hairpins [33]. In addition, the formation of β-sheets and β-strands can be induced by macrocyclization or even the introduction of amyloid-like fragments, which are known to self-associate by forming extensive β-sheet layers [33].

Although macrocyclic peptides can be considered valid therapeutic candidates for their improved affinity for a target and stability, they do not possess a proper balance of rigidity and flexibility and also lack the appropriate solubility required to move between aqueous and lipophilic environments [40]. To ameliorate this undesirable feature of macrocyclic peptides, it can be convenient to introduce, inside smaller cyclic peptides, residues provided with N-methylation and D-configuration. Such small cyclic peptides can be more suitable for oral dosing as they can be characterized by better metabolic stabilities with respect to macrocyclic peptides [40].

The bioavailability of peptide drugs, which is highly reduced by enzymatic cleavage occurring in the intestinal tract, can be enhanced by coformulations with inhibitors of protease activity. However, this approach may present side effects leading to digestion- and pancreas-related problems. It might be convenient to employ carriers that enhance peptide absorption; these conveyors include a variety of chemical molecules that can operate through several mechanisms, such as the opening of tight junctions and modulation of membrane fluidity and mucus viscosity [33,35]. Among the carriers, we can find chelating agents like ethylenediaminetetraacetic acid (EDTA) and citric acid, short fatty acids (i.e., molecules with an amphipathic nature) like sodium caprylate, sodium lauryl sulphate, or sodium taurocholate (representing an example of bile acid) [35].

A further route to improve peptide oral bioavailability and pharmacodynamic features consists of employing mucoadhesive polymeric systems (e.g., sodium carboxymethyl cellulose, polyacrylic acid, polyethylene oxide, methyl cellulose, etc.…) that become attached to the intestinal epithelium, thus improving absorption at the mucosal membrane [35,39].

Nowadays, the targeting of intracellular space with peptide therapeutics remains a great challenge [38,42,43]. From a historical perspective, peptide drug discovery was initially (through the 1970s) centered around receptor targets; later (1980s–1990s), remarkable accomplishments were achieved regarding the discovery of peptides showing good cellular permeability and capacity to regulate intracellular protein targets like the macrocyclic natural peptide product cyclosporin, and diverse groups of cell-penetrating peptides (CPPs) including the synthetic ones obtained by conjugation with the transactivating transcriptional activator (TAT). To describe in depth the methods to improve peptide permeability and delivery inside cells is beyond the goal of this work, as many reviews have been centered on the topic [42,43,44,45]. However, it is worth noting that several experimental and computational screening instruments to investigate peptide permeability and support the development of intracellularly targeted peptide therapeutics are presently available. For instance, there exist methods that implement cellular monolayers to analyze transcellular permeability; computational/biophysics tools comprise those focused on exposed polar surface features, the comparative analysis of intramolecular and solvent H-bonding by NMR and mass spectrometry (MS) analyses, the evaluation of octanol/water phase partitioning to establish energy-independent translocation, and the analysis of the radius of gyration, which represents a sort of substitute characteristic for the molecular weight in classes of molecules that do not conform to Lipinski’s rule of 5 [38,46].

To generate more drug-like agents, bifunctional systems, exploiting advantageous features of both peptides and small molecules, can be designed and evaluated with the support of in silico tools [47,48]. Indeed, once selected, a peptide with an established high affinity for a specific protein target and for which the 3D structure of the protein–peptide complex is available can be split into two fragments provided with a reduced (or not even appreciable) interaction affinity for the protein target. Next, reactive groups can be inserted at one terminal side of each fragment to allow for the exploitation of “click chemistry” to combine the two peptide segments with small molecules. In the end, screening can be conducted with a plethora of small molecules to find out which moieties are able to substitute one of the two peptide segments and restore the high affinity of the starting peptide [47].

The following sections will be focused on the diverse computational approaches for the development of peptides able to hamper crucial PPIs involved in pathological conditions. Several examples of in silico identified peptides or peptide-based molecules that can be considered promising starting points to develop therapeutic agents will be highlighted.

## 2. Identifying In Silico Novel Bioactive Peptides: Methodological Aspects

A variety of virtual screening approaches can be applied for the identification of novel potential bioactive peptides. Much attention needs to be given to peptide libraries to be implemented in such computational screenings, as they need to be designed by considering key peptide features that might influence bioactivity and drug-likeness [including, but not limited to, cell permeability, aggregation tendency, and stability (See Section 1.2)].

### 2.1. Design of Virtual Peptide Libraries

The construction of virtual peptide libraries for specific drug discovery applications can be supported by in silico tools to predict peptide bioactivity and drug-like characteristics. However, a mixed computational and experimental approach, which was undertaken to analyze bioactivity in a group of peptides derived from goat casein hydrolysate, highlighted that in silico bioinformatics tools to predict bioactivity have some limitations in terms of the range of activities that can be associated with peptide sequences and should only be used in combination with in vitro and/or in vivo tests. The disagreement in the results from diverse in silico predictors is also another potential drawback [49].

Moreover, numerical descriptive vectors (NDVs) for peptide sequences might be considered when planning which peptides are to be included in a library. Indeed, amino acids’ physicochemical features can be exploited to obtain, through principal component analysis (PCA), NDVs for peptide sequences [50]. NDVs possess a length equal to peptide sequences, and each entry in an NDV corresponds to an amino acid. NDVs can be employed for quantitative structure–activity relationship (QSAR) analyses of peptide groups and for the prediction of peptide residues representing hot spots [50].

A variety of virtual peptide libraries can be generated through diverse approaches depending on specific peptide properties that need to be satisfied to address the scientific problem under investigation, and a few examples will be provided below.

First of all, to support virtual screening approaches and speed up the computational identification of bioactive peptides, Prasastry and Istyastono provided the structures of 168,400 peptides (di-, tri-, and tetra-peptides) that were generated from all possible combinations of the 20 natural amino acids. The structures were reported in both a simplified molecular-input line-entry system (SMILES) and three-dimensional (3D) formats suitable for diverse molecular docking pipelines [51].

In addition, a powerful instrument for the fast computational analysis of peptide libraries is PDAUG (Peptide Design and Analysis Under Galaxy). Indeed, PDAUG includes several tools for the development of peptide libraries, data visualization, the calculation of peptide characteristics, modeling, and links to databases from which to retrieve peptide sequences. For instance, PDAUG includes the routine “amino acid Property Based Peptide Generation” that creates peptide sequences relying on amino acid properties. The “Sequence Based Peptide Generation” routine instead allows for the creation of peptide sequences through three diverse routes: “Random Peptides” (a method that seeks all the probable combinations of 20 natural amino acid residues within the chosen sequence length), “Mutated Peptides” (a method that, at specific positions, substitutes the existing residues with the residual 19 amino acids) and “Sliding Window Peptides” (this tool uses a protein sequence as input and randomly produces peptide segments based on a sliding gap and fragment dimension) [52].

Ad hoc-generated peptide libraries can be implemented for the design of PPIs by preserving specificity that generally characterizes naturally occurring interactions. In this context, a clever strategy made use of a library composed of 1536 peptides where 32-mer sequences were generated by including characteristic features of the parallel dimeric coiled-coil pattern along with the semi-random introduction of residues in positions crucial for stability and specificity. Computational screening was achieved to identify, based on the predicted Tm (melting temperature) values, eight peptides that could interact together by creating four heterospecific PPIs [53]. A combination of experimental techniques was, in the end, used to validate the capacity of this protocol to predict the formation of heterospecific PPIs. This computational approach provides information that can be applied in different fields of protein science, including the discovery of therapeutic peptides able to specifically inhibit PPIs relevant to certain pathological conditions [53].

Degradation by proteases is one of the challenges that is faced when attempting to develop therapeutic peptides (See Section 1.2). A valid solution relies on the screening of peptide libraries containing D-amino acids; however, experimental strategies based on such an approach are generally time consuming and expensive due to the costs associated with the synthesis of peptides containing the unnatural D-amino acids. In silico screening campaigns with virtual libraries of D-peptides can be planned to ameliorate issues associated with the experimental methods. An interesting approach in this field is exploited by the web server finDr (https://findr.biologie.uni-freiburg.de/) that supports the identification of the D-peptide interactors of target proteins. finDr works similarly to a mirror-image phage display where the peptides are assayed in the L-configuration while the target is produced in the D-configuration. If an L-peptide interacts with the D-form of a protein, its mirror images (D-peptide) will be able to interact in a similar manner to the same natural protein in the L-configuration [54]. Starting from this concept, the virtual library employed by finDr consists of helical peptide fragments (12 amino acid long) extracted from Protein Data Bank (PDB) entries. Such a library is screened against the chosen protein target in the D-configuration through Mirror-Image Virtual Screening (MIVS). This leads to a prediction by molecular-docking of L-peptide ligands, the configuration of which can be inverted to obtain D-peptides interacting with the naturally occurring L-protein [54].

The bioactivity of short peptides can be modulated by their ability to self-assemble into supramolecular structures. Another interesting study reported on the employment of a library of 400 dipeptides, representing all possible combinations of natural amino acids, which were subjected to a coarse-grained molecular dynamics approach to predict their capacity to self-associate [55].

Bioactive peptides hold great interest in drug discovery; however, different to small molecules, for which a vast chemical space can be explored, peptide optimization can only rely on the reduced set of natural and commercially available non-natural amino acids [56]. To overcome this limitation, recently, a large virtual library of non-natural and readily synthesizable amino acids was developed to enhance virtual screening approaches and assist the peptide optimization phase. The library was generated starting from the eMolecules database, that includes more than 26 million compounds. This database was first analyzed to select the included α-amino acids. Moreover, the eMolecules database already contains non-natural amino acids, but to expand this group, chemical reactions were simulated based on a few synthetic canonical routes. To overcome selectivity issues, the compounds in the eMolecules database provided with more than a single reactive group were excluded from the approach to avoid several side products. The compounds leading to reaction products containing more than a single α-amino acid group were automatically excluded as their inclusion in peptide sequences could be challenging. The compounds were included in the final library only if certain rules were respected, concerning, for example, the molecular weight, number of chiral centers, number of heavy atoms, formal charges, etc.…. Once a target protein–peptide complex has been chosen, the library can be implemented to set up the in silico optimization approach. Modeling studies can be conducted to insert the non-natural amino acid members of the library in the crucial positions of the natural peptide ligand, and docking studies can predict improved interaction affinity and specificity [56]. This strategy can support the selection of the most promising peptide ligands to be synthesized and experimentally validated.

Virtual screening represents a powerful approach when a peptide ligand of the target protein has already been identified and validated, but it needs to be optimized. For such computational screenings, the use of libraries of peptides provided with good predicted affinities and specificities for the target protein should be preferred with respect to randomly generated mutant peptide libraries. A library optimization method that relies on experimental binding information or in silico produced structural data to guide the best amino acid substitutions at each peptide position has been proposed [57]. Once a protein–peptide target system has been chosen, the method can exploit diverse data, including experimental binding information derived, for example, by SPOT arrays and alanine scanning, if available, or in silico-generated scores for peptide mutants if a 3D structure of a protein–peptide complex has been solved [57]. This information is implemented to classify amino acid substitutions into two sets: “required” and “preferred”. “Required” mutations will constantly be incorporated when developing a new library and comprise the amino acids of the starting wild-type peptide sequence along with additional residues, the influence of which on the affinity and specificity is proved by experimental or computational data. The “preferred” group will include substitutions that an optimization algorithm identifies as possible instead [57]. After initial library generation, further analyses and iterative optimization steps can be completed [57].

Another active area of research focuses on the design of cyclic peptide libraries for in silico strategies. As explained in Section 1.2, cyclization is another route to enhance the peptide drug-likeness by reducing susceptibility to enzymatic degradation. Golosov and collaborators [58] described the design of peptide libraries containing a macrocyclic arrangement to allow for both permeability and oral exposure [58]. Such a library was built by speculating that a properly chosen N-methylation pattern that could favor passive permeability should stabilize a macrocyclic structural organization provided with a low desolvation penalty for the passage from water to a membrane-like milieu [58]. Different libraries of macrocyclic peptides were designed (i.e., 6-mer, 7-mer, or 8-mer members), and macrocyclization was achieved through a thioether bond, as it could be experimentally easily generated upon a reaction between the side chain of a C-terminal cysteine and an N-terminal electrophilic group [58]. Moreover, other rules were considered to generate peptides: the presence of a UV chromophore (i.e., D- and L-phenylalanine with or without N-methylation), presence or absence of a proline residue, presence of mimics of the leucine residue (i.e., norleucine and backbone methylated norleucine), as leucine is contained in certain oral natural products and peptides, and a maximum of three or four N-methylated residues [58]. After the initial design, the in silico generation of peptide models was achieved along with conformational sampling in an environment characterized by a low dielectric constant to simulate the nonpolar membrane region. Next, transfer-free energies for the passage from an aqueous to a membrane-like background (ΔG_transfer_) were estimated for each peptide conformation. This phase was followed by the evaluation of those conformations characterized by both low energy and low ΔG_transfer_ values for each virtual peptide. These combined energetic considerations were employed to guess potentially permeable macrocycles (i.e., those provided with low ΔG_transfer_ values and conformations with the reduced number of solvent-exposed backbone amide protons). Indeed, the best-selected macrocyclic peptides were synthesized and in vitro tests proved the effectiveness of this screening protocol. This approach can be nicely applied for the identification of sets of permeable cyclic peptides characterized by large side-chain diversity [58].

Peptidomimetic macrocycles are very attractive in drug discovery not only for their drug-like character but also for their potential ability to target the most undruggable protein targets. One limitation of drug discovery based on peptidomimetic macrocycles is often related to difficult synthetic routes. Saha and collaborators recently created a computational platform to generate libraries of synthesizable peptide macrocycles deriving from a multistep reaction series [59]. The platform relies on previously established experimental protocols to merge small linear peptides with synthetic scaffolding reagents. The merged bifunctional compounds are next converted into amphipathic macrocycles provided with specific conformations and enhanced pharmacological features. This computational tool is made up of two principal components: the “composite peptide macrocycle generator” (CPMG) and “ConfBuster++”. CPMG can be first employed to create, starting from user-selected building blocks, a library of two-dimensional (2D) macrocycle structures; the conformations of each macrocycle are next produced by ConfBuster++ [59]. In detail, the library can be created starting from an ensemble of building blocks made up of amino acid derivatives having diverse drug-like and conformationally constraining patterns. Linear oligopeptides are produced by CPMG through methodical permutations of building blocks and are then inserted into specific templates. Template-linked oligopeptides are subsequently transformed into macrocyclic arrangements following rules gained from experimental studies and considering estimates for site reactivity. To favor maximal diversity in macrocycles, a filtering strategy can be applied based on the analysis of physical and three-dimensional characteristics. This in silico approach represents an attractive tool to be considered before starting very large virtual screening campaigns with macrocyclic peptides/peptidomimetics to favor the selection of peptides that could be more easily synthesized by established routes [59].

When planning the design of a peptide library for virtual screening, it is also important to evaluate the possibility of introducing, within peptide sequences, cell-penetrating motifs. Cell-penetrating peptides attract attention for intracellular delivery as they can work as carriers for different classes of molecules (e.g., small compounds, peptides, and oligonucleotides), thus overcoming their low bioavailability issue. In this context, computational tools to design CPPs can be very supportive [60,61]. The free web tool “CellPPD” (i.e., http://crdd.osdd.net/raghava/cellppd/) can be, for example, implemented to predict CPPs with elevated accuracy [60]. The approach behind “CellPPD” relies on the exploitation of different peptide features (e.g., the type of residues in each position, two-residue motives, and physicochemical properties) to develop support vector machine (SVM)-based models through which it is possible to discriminate between CPPs and non-CPPS [60]. The likelihood of false positives is a problem that can be encountered in CPP prediction, and the ”AiCPP” approach seems to provide a possible solution [61]. In detail, this is a deep learning-based method employing a negative control group made up of a vast set of peptide sequences from human reference proteins [61].

In silico tools to generate peptide libraries able to hamper the binding of antigens to antibodies have also attracted attention. Synthetic peptides constitute a good alternative to antibodies that, although holding great therapeutic potential, might be characterized by unfavorable drug-like features. In this field, the “epitope-paratope interaction (EPI)-peptide” designer tool can be considered a promising computational instrument. Its workflow consists of the initial identification of interacting residues within the contact interface of an antibody–antigen complex through the selection protocol “Interface Interacting Residue (I2R)” [62]. Based on the calculated ensemble of all the intermolecular contacts, the 3D structure of the antibody–antigen complex is translated into interface diagrams. In the end, antibody residues, predicted to bind to the target epitope, are exploited by the EPI-Peptide Designer tool to develop libraries of peptides that should work as ligands of the antigen under investigation and thus possibly function as therapeutic agents [62].

Similarly, a virtual screening method to iteratively develop virtual libraries of peptide ligands able to target the Fc portion of the IgG (Immunoglobulin G) antibody will be described in the next paragraph [63].

Instead of employing very large virtual peptide libraries for virtual screening, it can also be convenient to explore the “in silico panning” approach to identify a peptide ligand of a target protein starting from a small library by merging docking studies with genetic algorithms (GAs) through which peptide ligands are iteratively developed [64]. For “in silico panning”, docking analyses of a small peptide virtual library are conducted first so that peptides are ranked according to their docking scores and predicted affinity [64]. Then, the best peptide ligands are evolved through GAs. GAs somehow simulate the genetic evolution of biological systems and include three steps (selection, crossover, and mutation) that are carried out on an ensemble of sequences to produce a novel peptide generation. The process is repeated several times, and during evolution, the docking energies decrease, leading to the identification of optimized peptide ligands. Yagi and collaborators reported on the application of “in silico panning” to identify a non-competitive inhibitor of the water-soluble quinoprotein glucose dehydrogenase starting from a virtual peptide library composed of just 10 tetrapeptides including seven diverse amino acids and performing four rounds of selection [64].

Interestingly, GAs are also key elements of another protocol developed to avoid the molecular mass repetitiveness that can characterize the combinatorial construction of peptide libraries. In detail, multi-objective GAs have been used to generate large libraries by ensuring the maximum possible number of permutations between positions of peptide sequences and a low probability of having library members with identical masses and/or shared sequences [65].

### 2.2. Virtual Screening Approaches in Brief

Peptides attract attention for their involvement in the regulation of a considerable number of biological processes, as peptide epitopes mediate an array of PPIs [66]. The size and flexibility of peptides allow them to interact with high specificity to diverse receptors, even to those considered undruggable from the perspective of small molecules. In addition, the potential capacity of peptides to interact with large and flat protein surfaces makes them ideal candidates as therapeutics able to inhibit PPIs that are crucial in modulating pathological conditions [66].

Furthermore, certain peptides can self assemble, forming hydrogels, fibers, and a variety of structures that can find several applications in biomedicine, even as supports for tissue regeneration.

Computational routes are surely helpful and convenient for the prediction of bioactive peptides possessing a particular self-assembly ability or capacity to bind with high affinity and specificity to a protein target and block crucial PPIs [66]. In drug discovery, virtual screening can quickly support the identification of the most promising peptide hits that should be first synthesized and submitted to experimental validation to speed up the overall process and also reduce connected costs.

Virtual screening approaches can be subdivided mainly into two categories: structure-based virtual screening (SBVS), that relies on the complementarity between the ligand and the target’s binding pocket (i.e., receptor–ligand docking), and ligand-based virtual screening (LBVS), which instead employs active ligands as prototypes to target protein receptors. LBVS methods are focused on the selection of candidate molecules, considering that a peptide interactor similar to a bioactive peptide ligand has a higher chance of possessing biological activity. A computational route combining both ligand and receptor-based approaches is often a good choice to identify original molecules and reduce the chance of finding false positives [67].

Challenges usually associated with interactions involving peptides include a low amount of available 3D structures and a lack of information on the binding affinities linked often to the transient nature of peptide-based interactions. In addition, the intrinsic flexibility of peptides highly affects the success rate of computational tools for structure and affinity predictions [66].

Molecular docking predicts the structural arrangement of the ligand, as well as its orientation and position at the binding site [68]. Two diverse groups of docking algorithms to predict protein–peptide interactions exist: template-based and template-free [69]. The template-based approach employs the 3D structure of similar complexes as a model to guess the binding mode of a peptide to a protein, and GalaxyPepDock [70] is an example of such a docking tool. The application of template-based docking has certain limitations related to the restricted availability of templates. No template is required for template-free docking, which includes two diverse approaches (i.e., global and local docking) that can be applied to different case studies depending on whether the knowledge of the binding pocket is available or not. Global docking samples the total protein surface to obtain the peptide interaction mode and identify the binding pocket. Instruments to achieve global docking include the webservers pepATTRACT [12] and HPEPDOCK [71].

Local docking relies instead on a user-defined binding pocket around which to look for peptide binding poses. Popular programs for local docking are AutoDock Vina [72,73], GOLD (Genetic Optimisation for Ligand Docking) [74,75], and HADDOCK (High Ambiguity Driven protein-protein DOCKing) [76,77]. A larger list of docking software for protein–peptide complexes can be found in a 2020 work by Weng and colleagues [69].

Three main subgroups of docking approaches exist: rigid, flexible, and semi-flexible [68]. In rigid docking, ligands and proteins represent rigid objects, and sampling is achieved by keeping in account only six degrees of freedom (i.e., three translational and three rotational ones). This approach can be mostly implemented for protein–protein docking, as in this case, the total number of conformational degrees of freedom are too many to be sampled. Instead, in semi-flexible docking, the conformation of the receptor or peptide ligand can undergo some fluctuations during docking runs. Usually, flexibility is contemplated for ligands as they have a lower number of conformational degrees of freedom that can be sampled during docking runs. Such an approach considers the rigid structure of the receptor as the conformation able to interact with the peptide ligand. Flexible docking assumes instead flexibility in both the protein and ligand and is generally based either on an induced fit interaction model or on conformational selection [68].

The DINC (Docking INCrementally) 2.0 and iMolsDock web tools represent valid examples of computational instruments to face the flexibility issue which, as mentioned before, can characterize not only peptide ligands but also protein receptors [78,79].

Hence, protein–peptide complexes can be studied using different pipelines depending on the presence of flexibility in the protein alone, in the peptide alone, or in both [79]. When dealing with flexible ligands rather than small molecules, it needs to be kept in mind that virtual screening by molecular docking might possess limited accuracy for ligands characterized by more than 10 flexible bonds [78]. Interestingly, the protein–peptide docking webserver named DINC 2.0 moves this limit to 25 flexible bonds thanks to a parallelized meta-docking approach through which large ligands can be docked against target proteins in an incremental manner [78]. Rather than performing a docking of the entire ligand, the complexity is reduced by incrementally docking larger and overlapping sections of the ligand. Indeed, in the first step, the docking of a small portion of the ligand with only six degrees of freedom (DoFs) is achieved, and the best binding modes are selected [78]. Starting from the selected docking poses, the related fragment is amplified through the addition of ligand atoms; afterwards, a new docking round is carried out. Successive docking runs are conducted, and every time, three new DoFs are included and combined with the previously considered fragment for which just three flexible DoFs are maintained [78]. Therefore, at each docking round, independently from the fragment dimension, only six internal DoFs of the ligand are considered. This stage is repeated until the entire structure of the peptide ligand is covered and docked [78].

An induced-fit docking instrument called iMolsDock employs the mutually orthogonal Latin squares (MOLS) sampling method and is able to consider protein flexibility [79]. A recently updated software version allows for enhanced receptor flexibility, better scoring function, and fast calculations. Initially, the MOLS approach was used to perform peptide modeling by relying on the concept that (S)^k^ conformations exist for a peptide provided with k torsion angles that can assume s diverse values. The conformational space that can be sampled by the peptide derives from the contribution of all possible combinations of torsion angles. The MOLS approach is able to guess the optimal peptide structure with the lowest energy by inspecting the peptide conformational energy landscape. MOLS was then expanded to perform docking. The ligand docking pose can be obtained by the tool iMOLSDOCK through ligand conformational sampling, including rotation and translation. Nevertheless, to keep protein flexibility into account, the search space of the docking routine was extended to include the conformational space accessible to flexible residues in the receptor [79]. The induced-fit docking protocol of iMOLSDOCK assumes that if the conformations of the ligand and flexible residues in the protein rely on k and l torsion angles, respectively, and the interaction pose of the ligand is established through six extra parameters (three related to the position of the ligand and extra three related to its orientation in the protein binding site) then, the space that is sampled will have k+l+6 dimensions with a volume equal to (s)^k+l+6^ and every dimension will be sampled s times. In the end, to find the optimal binding pose for the ligand in the protein interaction pocket, a gradient minimization is performed as well [79].

Among the protein–peptide molecular docking instruments, it is worth mentioning CABS-dock. CABS-dock exists as a webserver and a separate program; it represents another approach for docking that can keep into account a substantial contribution from conformational flexibility for both the protein and peptide [80]. CABS-dock does not involve a predefined binding pocket and considers the peptide as fully flexible. Concerning the protein, its backbone can be subjected to small fluctuations and optionally larger movements [80].

For flexible ligand receptors, another appealing docking web tool is HTP SurflexDock. This is a docking instrument aiming to enhance SBVS’s success rate by combining two routes. In the first stage, the ensemble docking protocol is implemented to simulate the inherent receptor flexibility. In the second step, the best docking hits can be rescored either through a search of a larger conformational space or by evaluating the interaction-free energy by means of the molecular mechanics (MM)/Poisson–Boltzmann surface area (PBSA) method [81].

PepVis was instead developed with the goal of making available to the scientific community an effective instrument to automate peptide virtual screening by combining several open-source tools to carry out ensemble and flexible docking approaches [82]. Virtual screening through the Graphical User Interface (GUI)-based pipeline PepVis is achieved by exploiting a combination of several docking tools (i.e., AutoDock Vina [72], ZDOCK [83], and AutoDock CrankPep [84]) [82]. In addition, this pipeline relies on two bioinformatics tools for the modeling of peptides (i.e., Modpep [85] and GROMACS (GROningen MOlecular Simulation) [86,87]), one for the re-ranking of peptides (i.e., ZRANK2 [88]) and one for the refinement of the receptor–peptide complex (i.e., FlexPepDock [89]) [82]. The advantage of this pipeline is its modular nature, which allows for the insertion of additional bioinformatics tools, thus increasing the information that can be acquired through it [82].

As mentioned earlier, a virtual screening protocol specific for the identification of peptide ligands able to target the Fc portion of the IgG antibody has been developed; such an approach is also very useful for virtual library development [63]. In the first step, a combinatorial library of tetrapeptides was designed by combining the 20 natural amino acids, and the conformations for each peptide were generated so that the 3D structures were available for each library member. The peptide library was screened against the crystal structure of IgG1 Fc by the docking software CmDock (CurieMarieDock) (v. 0.2.0) [63]. In addition, the best predicted ligands (i.e., 100 tetrapeptides) were subdivided into clusters and further analyzed to estimate the residues important for interaction with the antibody and determine the highest occurrence of specific amino acids in each peptide sequence position [63]. Interestingly, the CmDock program identified preferred amino acid positions inside the top-scoring peptides, which were consistent with the current knowledge of peptides binding to the Fc region of IgG. The method can be further implemented to develop a more focused library for the identification of peptide ligands of antibodies with extended sequences [63]. Indeed, the protocol could proceed iteratively and even be used for developing focused libraries against different targets rather than antibodies.

Instead, a kind of inverse virtual screening approach was set up based on the “select and purge” (SP) algorithm by combining sampling by statistical approaches and selection through rigid molecular docking. This virtual route allows for the identification of short peptides able to function as receptors for small compounds when no knowledge about the peptide/small molecule association is available [90]. Briefly, this approach requires the assembly of a library of small molecules by considering different criteria for compound selection [90]. Concerning the small peptides to be investigated, tripeptides deriving from all possible combinations of the 20 natural amino acids can be implemented. Then, docking analyses are conducted to predict the binding affinities for the peptides/small molecule complexes [90]. The resulting data are exploited by the select and purge (SP) algorithm to further assess how amino acid positions and types can influence the ability of tripeptides to form complexes with specific ligands [90]. False positives are eliminated, and the best candidates are chosen based on the unfavorable and favorable (i.e., those stabilizing the complex) pairs of amino acid positions within the analyzed peptide sequences. The algorithm works iteratively, and after the stop point is reached, a set of possible peptide receptors is detected among the investigated dataset [90].

Specific in silico pipelines, somehow different to the common docking-based virtual screening protocols described before, were applied to cyclic peptides [91,92]. As mentioned before, constrained bioactive peptides assume particular relevance in drug discovery due to a likely increased specificity for the target and higher resistance against proteases [91,92]. The cyclic PEPtide matching (cPEPmatch) method consists of a fast in silico route to identify cyclic peptides that could interact with specific segments located at the interface of protein–protein complexes [92]. This approach is based on the comparison between the backbone of short peptide fragments, derived from the protein–protein binding surface, and the backbone structures of an ensemble of cyclic peptides. When a positive match is found, the corresponding cyclic peptide is taken as a model interactor and used as a template by adjusting its amino acid side chains based on those of the protein–protein complex. For 154 protein–protein interactions that were analyzed against a small library of cyclic peptides for which structures were available, at least one peptide template could be found for a specific region of a protein–protein interface. Interestingly, for most of the predicted cyclic peptide ligands, MD (molecular dynamics) analyses and binding energy evaluations revealed stable protein–cyclic peptide complexes and good binding energy scores, thus indicating the promising effectiveness of the method [92].

It is not easy to establish which protein–protein interface is suitable to be targeted by cyclic peptides. A high-throughput approach and a rapid method for the virtual screening of peptide libraries were set up by Duffy and colleagues [91]. First, combinatorial 3D peptide libraries, including small peptides containing disulfide bridges, were developed in silico. Next, diverse members of these libraries were compared and matched with pharmacophore models based on the structures of crucial protein–peptide and protein–protein complexes. These pharmacophores were constructed by analyzing the 3D structures available in the PDB and consisted of short peptides known to bind proteins, linear motifs, and turns characteristic of protein–protein interaction surfaces. In the end, pharmacophoric models were compared with more than 100,000 cyclic peptides in multiple conformations [91]. This pharmacophore screening is a protocol useful to predict not only the PPIs suitable to be targeted but also the cyclic peptide modulators of these optimal PPIs that can be thus prioritized for synthesis and experimental validation [91].

#### Limitations of Virtual Screening Approaches

SBVS of compound libraries is a widely employed approach at the initial stages of the drug discovery process to support hit-finding campaigns [93]. Indeed, high-throughput virtual screening and molecular docking platforms have become very popular within the scientific community due to their great capacity to analyze large databases of molecular entities with low costs and rapidity [94]. The employment of protein–ligand docking algorithms to identify potential modulators of disease-related proteins has also been supported by the increasing number of high-resolution protein structures being solved by X-ray crystallography and other experimental techniques, the advancements in molecular modeling platforms, and the development of very powerful computer resources [95].

Molecular docking represents the key point in SBVS [96]. Briefly, it includes two principal phases relying on two diverse algorithms. The diverse conformations (i.e., poses) that a ligand can assume in the receptor binding site are predicted by the sampling algorithm. In addition, the binding energies associated with the receptor–ligand poses are predicted by a scoring function. Scoring functions are generally used to achieve the filtering and ranking of the obtained poses in virtual screening, indicating which screened compounds should represent a possible lead [96]. A robust virtual hit selection is particularly important when working with new targets and can rely on a variety of protocols, including scoring, ensemble docking, consensus pose, and ligand efficiency, to enhance the accuracy of docking results [97]. Due to improvements in computer resources and in the dimension of compound libraries, it is possible to screen billions of molecules with computer clusters provided with a modest size [93]. However, the fast exploration of such a large chemical space though VS requires approximations, leading to the under-sampling of potential configurations and incorrect estimates of absolute interaction energies [93]. Consequently, the usage of docking-based VS alone comes with faults. Nevertheless, different laboratories plan VS workflows based on software and techniques they are more familiar with, but also, when software appears simple to use, every practice presents several weaknesses that need to be considered to avoid incorrect results/artifacts [98]. Moreover, the simplification of molecular docking lowers the efficiency of the docking score, leading to a higher chance of obtaining false positives [99].

As briefly mentioned in Section 2.2, one major issue of VS is often related to the static nature of the receptor during rigid docking, where the dynamic features of biological structures are not properly taken into account [96]. For practical reasons, most docking platforms employ a rigid receptor approximation, in which the ligand is considered a flexible entity, while the conformation of the protein is constrained. A few approaches, like ensemble docking, can be instead a better choice as, in this case, an ensemble of receptor conformations allows for the investigation of protein flexibility [99]. Nevertheless, the docking of large peptidomimetics is challenging with ordinary docking protocols (see also Section 2.2) as docking algorithms can be rather inaccurate in predicting the right poses for molecular entities provided with many rotatable bonds. In fact, the addition of each rotatable bond induces an enlargement of the conformational space to be sampled and a decreased chance to obtain the correct binding pose [96].

The generation of both false positives and false negatives by docking-based VS can be due to limitations in sampling algorithms and flaws in scoring functions; in addition, the need for training groups in diverse algorithms frequently induces a highly target-dependent accuracy [96]. Another drawback is linked to docking poses and scores severely subjected to the ligand input conformation. In fact, subtle variations in the ligand input structure can induce significant divergences in the resultant docked poses [99]. Much care needs to be taken in the initial preparation of the ligands and structures to be submitted to VS [98].

A significant limitation of the docking software is often linked to the inability of scoring functions to correctly predict ligand binding affinities [99]. Consequently, the best docking hits should not be selected just based on energy scores. Docking scores should be used to discard molecules that do not fit the binding/active pockets but not to establish thresholds for biological activities. Another aspect that needs to be crucially considered is that the comparison of docking scores from diverse docking platforms is not reliable, as these scores are strictly linked to the force fields and protocols implemented by each software [99]. Another important aspect of molecular docking is related to the specific treatment of water molecules that are present inside the binding pocket throughout the docking progression, that could induce the erroneous evaluation of the potential interactions between the receptors and ligands [99]. Finally, the capacity of docking software to distinguish between inactive and active molecules is generally largely connected to the employed protein structure and the degree of similarity between the screened compound and a co-crystallized ligand [99].

As mentioned before, a key issue is the failure of scoring functions to properly rank compounds based on their activities towards the chosen target protein [95]. There are several factors that might negatively impact the performance of scoring functions in successfully guessing interactions energies. Empirical functions deliberately have a simple form in order to allow for computational speed, and can often only slightly compensate for the rigid protein approximation. In addition, several penalty terms, including electrostatic and steric clashes and internal ligand constraints, cannot be easily and correctly parameterized. Due to several approximations, scoring functions can be too “soft”, leading to many incorrect hits (i.e., false positives) [95]. Moreover, in the context of scoring functions, it needs to be recognized that entropy and desolvation are problematic to be handled even inside a strict molecular mechanics formalism [95]. The limitations and difficulties of using molecular docking in nutraceutical research have been covered in a recent work by Agu and coworkers, which also discusses the reliability of scoring functions and the requirement for experimental validation [100]. Limitations in docking-based VS to identify compounds targeting SARS-CoV-2 (Severe Acute Respiratory Syndrome-Coronavirus-2) proteins have also been reported in a few works [97,99].

It emerges from the literature analysis that precautions need to be taken before starting HTVS studies to identify biologically active compounds. For example, regardless of the employed docking platform, it is important that VS is conducted through several trials and that docking solutions are visually inspected to observe similarities in binding poses and recognize artifacts [94]. The reproducibility of the docking experiments should be assessed through the analysis and validation of interaction poses referring to a minimum number of trials, and this protocol also ensures the identification of the correct protein–ligand structural topology of binding [94].

If structural data can be retrieved from the Protein Data Bank (PDB), the docking results should be, of course, further validated through comparison to high-resolution experimental structures of protein–ligand complexes [94].

It is also recommended to validate results generated through HTVS further by molecular dynamics simulations, where the dynamics of both the ligand and receptor are allowed, and the stability of the interaction poses can be better estimated [94].

Within the context of VS, re-scoring is something to keep in mind, as this can yield an improved correlation between the docking results and experimental evidence [96]. For the re-scoring of docking solutions, standard molecular dynamics can be coupled to the prediction of binding free energy through MM-GBSA (molecular mechanics with generalized Born and surface area solvation) and MM-PBSA (molecular mechanics/Poisson–Boltzmann Surface Area) [99]. MM-GBSA or MM-PBSA are usually associated with modest computational efforts; they produce more solid results than those provided by most docking scoring functions and have been largely exploited by the scientific community. These computational techniques do not represent tools for screening large libraries of molecular entities but are useful for analyzing the selected docking poses [99]. Coupling molecular docking with MM-GBSA or MM-PBSA re-scoring can be considered a very promising approach to selecting the correct binding poses and ordering a group of ligands according to the binding affinities. Again, the success of these approaches is target structure dependent [99].

The availability of a lot of published data on the protein target increases the chance of achieving a successful VS campaign [96]. The accessibility of the high-resolution X-ray or NMR structures of the receptor is also vital before starting the in silico screening, as a reduced performance of VS could be achieved by using homology models [96]. Nevertheless, if possible, the employment of docking-based VS of holo-structures, rather than apo- ones, could further enhance the success of in silico approaches leading to the enrichment of promising hits. Exhaustive knowledge of the active and/or allosteric interaction pockets, including a complete picture of the flexible side-chains and their positioning within the binding site, the occurrence of water molecules inside binding pockets, and the exact protonation forms of ionizable residues, will also contribute to the positive outcome of a VS campaign [96].

As VS protocols include several computational techniques, in the end, virtual screening hits, including those validated through MD and binding free energy calculations, represent just predictions that require further in silico and experimental testing to validate their activities. In silico testing can be achieved by running additional parallel virtual screenings of groups of known active and decoy molecules, if available [98]. Experimental in vitro and/or in vivo validation of the most promising computational hits must be performed after the proper compound selection has been achieved. The selection of biologically active ligands (such as possible therapeutic agents) by VS might also take ADME and solubility predictions into account [98]. The experimental validation of the predicted active VS hits should not be considered the end point of the drug discovery process as the newly identified compounds might represent just the base for further optimization cycles [98].

Practical guidelines that can be followed for large-scale docking have been reported in an interesting work by Bender et al. [93]. The authors describe the best practices to follow before starting a massive docking-related VS approach and suggest the software-independent controls that are needed to choose the best docking parameters for a specific target receptor and all the required controls to possibly obtain hits with desired activities, once experimentally validated [93].

## 3. Identifying Novel Bioactive Peptides through In Silico Approaches: Applications

The critical role played by in silico tools for the discovery of novel bioactive peptides has been proved in different biomedical fields, such as the development of therapeutics against cancer or viral infections, immune system regulators, antidepressants, anxiolytics, analgesics, as well as peptides for the treatment of nicotine addiction, hypertension, Parkinson’s disease, and neuropathic pains [101,102,103,104,105,106,107,108,109,110,111,112,113,114,115,116,117,118,119].

### 3.1. General Overview

The first step common to all diverse computational strategies consists of choosing a target protein or PPI for the specific class of bioactive peptides to be identified.

In searching for antihypertensive drugs, the angiotensin I-converting enzyme (ACE) can be employed as a target and the computational screening of peptides deriving from the hydrolysis of proteins extracted from animal sources is a potential approach [101]. A mixed in vitro–in silico strategy based on ACE has been reported; the experimental protocol included the in vitro hydrolysis of α-lactalbumin, the isolation of fractions with ACE inhibitory action, and the identification within these fractions of diverse peptide sequences that were employed to generate a peptide library. The library was next implemented for computational screening and docking poses for the ACE/peptide complexes, which were analyzed to predict peptides potentially able to hamper ACE activity efficiently. Docking studies were also valuable for distinguishing the competitive peptide inhibitors (i.e., those able to interact with the residues of the active site) from the non-competitive and mixed ones [101]. A diverse strategy to identify the peptide inhibitors of ACE started from the in silico hydrolysis of the protein nebulin from *Larimichthys crocea* to generate tripeptides that were subsequently employed in virtual screening from which the “HGR” (Histidine-Glycine-Arginine) sequence emerged as a promising bioactive agent [102]. Similarly, the “WCW” (Tryptophan-Cysteine-Tryptophan) peptide has been predicted as a possible ACE inhibitor using a fully computational approach based on the virtual screening of a tripeptide library containing 8000 peptides that was assembled considering that di- and tri-peptides, resulting from the enzymatic digestion of food proteins, might work to block ACE activity [103]. Due to the presence of antihypertensive peptides in diverse natural sources and the possibility to exploit them as therapeutics provided with reduced side-effects with respect to synthetic drugs targeting ACE, a web tool was even established through which antihypertensive peptides could be designed, selected by screening, or localized within a protein sequence [104].

Similar approaches were applied to Dipeptidyl-peptidase IV (DPP-IV), which can be considered another appealing therapeutic target as DPP-IV inhibitors can be effective in treating type 2 diabetes. For instance, a combination of peptidomics and computational studies allowed for the establishment of characteristic peptide features important in hampering DPP-IV activity. In this case, the peptide set screened in silico against DPP-IV was derived from a protein extract resulting from the in vitro digestion of Pinto bean [120]. The design of a virtual tripeptide library and virtual screening also led to the prediction of potential peptides inhibiting α-Glucosidase activity for therapeutic applications, as α-Glucosidase is linked to diverse pathological conditions, including diabetes mellitus 2 and obesity [115].

From the above-reported case studies, it is evident that the employment of peptide libraries derived from the in silico/in vitro hydrolysis of specific proteins or protein active fractions from natural extracts is a common strategy that can be employed to set up computational screenings. The in silico protein hydrolysis and virtual screening of the resulting peptides against a human receptor (i.e., the heterodimer made up of taste receptor type 1 member 1 (T1R1)/taste receptor type 1 member 3 (T1R3)) were even conducted to identify umami peptides derived from *O. mykiss* (i.e., rainbow trout) [121].

As mentioned before, selecting a proper target is critical [105,106]. For instance, in silico protocols with the purpose of providing novel potential antidepressants, anxiolytics, and analgesics can be set up based on the kappa opioid receptor (KOR), the γ-Aminobutyric acid (GABA)-A receptors, and the α2δ auxiliary subunit of V-gated Ca^2+^ channels (VGCCs) [105,106]. A computational study was planned to identify compounds targeting KOR by including drug design, lead optimization, and molecular dynamics (MD) steps [105]. Starting from the initial virtual screening of a library of 6 million structures, two tripeptides were selected as best hits. The tripeptides were synthesized and assayed in vivo, demonstrating a promising antinociceptive action [105].

One very active research field relies on the application of in silico techniques to identify peptides with anticancer potential (See below) [107,108,109,110]. For instance, Caseinolytic protease P (ClpP), Ets-like protein 1 (Elk-1), the programmed cell death protein 1/programmed cell death ligand 1 (PD-1/PD-L1) pathway, the polo-box domain of polo-like kinase 1 (PLK1-PBD) and interleukin-6 (IL6) modulate different processes (e.g., the degradation of misfolded proteins, gene regulation in response to extracellular signals, immune escape, proliferation, inflammation) linked to cancer onset and progression or acute myeloid leukemia (AML) and represent only a few possible targets [107,108,109,110,111]. The application of in silico methodologies alone or coupled to experimental validation with different direct and competition-type binding assays through an array of biophysical techniques has highlighted characteristic peptide features (e.g., the size and presence of non-natural amino acids) that are needed to appropriately target these specific proteins or related PPIs [107,108,109,110,111]. Another topic related to the anticancer drug discovery field, which is being widely explored in our laboratory through mixed approaches based on the computational screening of diverse in silico-generated peptide libraries and the experimental validation of selected peptide hits, consists of the identification of anticancer peptides/peptidomimetics targeting Sam (Sterile alpha motif)–Sam interactions mediated by the erythropoietin-producing hepatocellular receptor A2 (EphA2) [122,123,124].

Molecular docking techniques have also supported the identification of immunomodulatory peptides binding the Toll-Like receptor 2 (TLR-2) [112]. Major histocompatibility complex (MHC) proteins assume a crucial role in the field of adaptive immunity. Consequently, their interactions with peptides hold great interest and could be exploited to design vaccines, identify epitopes, and discover novel molecule regulators of the immune system. Doytchinova and collaborators set up an interesting in silico tool to analyze the protein–peptide binding affinity based on an additive quantitative structure–affinity relationship (QSAR) model that could be employed for the identification and optimization of peptides able to bind MHC proteins, and thus highly relevant for studies in the field of immunology [116,117].

A peculiar drug repurposing approach based on virtual screening was implemented for targeting the Calcitonin gene-related peptide receptor (CGRPR), which consists of a heterodimer involved in migraine insurgence and formed by calcitonin receptor-like receptor (CLR) and receptor activity modifying protein type 1 (RAMP1). The study aimed at identifying molecules able to hamper heterodimer formation and the results pointed out that pentagastrin, a synthetic polypeptide analogue of natural gastrin, could be further analyzed by experimental studies [114].

Among the challenges that can be encountered during virtual screening approaches, especially when dealing with peptides, the not always guaranteed availability of an experimentally derived structure for the target protein or complex and the presence of highly flexible regions, particularly within the peptides to be screened, represent two big issues. In this context, it is particularly difficult to target with in silico approaches the nicotinic acetylcholine receptor (nAChR), which is a protein involved in different diseases (e.g., nicotine addiction, Parkinson’s disease, and neuropathic pain). Thus, Leffer et al. proposed the docking algorithm ToxDock, that consists of an in silico tool based on the synergy between ensemble docking and extensive conformational sampling and performs well in the virtual screening of peptide ligands against an α4β2 nAChR homology model [125].

Another research area where in silico strategies are commonly implemented is the search for novel routes to cure Alzheimer’s disease (AD). To treat AD, one possible tactic could rely on the modulation of human serum albumin (HSA) carrier properties to remove as much as possible of the amyloid-β peptide (Aβ) from the central nervous system of patients affected by this pathology. Intriguingly, virtual screening strategies have been exploited as well to define HSA variants with the highest affinity for the Aβ peptide and gain information useful for the future development of therapeutics against AD [119].

This paragraph was intended to provide just a brief overview of the variety of settings in which in silico strategies were applied to search for bioactive peptides. Due to the large number of scenarios in which virtual screening and other in silico approaches can be applied, the next paragraphs will be centered more deeply on a few of the most appealing classes of bioactive peptides: anticancer, antimicrobial/antiviral peptides, and peptide inhibitors of fibrillogenesis. This choice is due to the relevance of these peptide groups in drug discovery focused on the most relevant pathologies [i.e., cancer, COVID-19 (Coronavirus disease 2019), AD, and Parkinson’s disease] for which the research is still struggling to reach an endpoint.

### 3.2. Anticancer Peptides

Cancer is characterized by the genetic damage of the cells causing uncontrolled growth and is accounted as one of the principal causes of mortality and morbidity worldwide [126]. According to the International Agency for Research on Cancer of the World Health Organization, breast, prostate, lung, and colorectum cancers are the most frequently diagnosed tumours affecting people of all ages (data from https://www.iarc.who.int/, accessed on 25 November 2023 [127]). Over the past few years, many drug discovery campaigns have been aimed at detecting efficacious anticancer agents. However, traditional cancer therapies, such as chemotherapy and radiotherapy, often are not selective to cancer cells or are unsuccessful due to cancer resistance [128]. On the other hand, more cutting-edge molecular targeting therapies, such as genome therapy and immunotherapy, based on an anti-tumoral molecule that is specifically directed towards unhealthy cells, have demonstrated limited effectiveness [129]. Therefore, alternative therapeutic approaches are needed, and in this context, anticancer peptides (ACPs) have great potential due to their high selectivity, their ability to penetrate cells, and the ease of chemical modification [129].

#### 3.2.1. Introduction

ACPs are small peptides often possessing a cationic property which confers them the ability to be selectively toxic to cancer cells characterized by negatively charged membranes. Most ACPs have similar characteristics to antimicrobial peptides (AMPs) because the surfaces of bacterial cells are also negatively charged; indeed, many AMPs are cytotoxic against both bacteria and cancer cells [128]. The structure of ACPs can be characterized by either an α-helical or a β-sheet conformation, but a few peptides with linear structures (lacking specific secondary structure elements) have also been reported. ACPs are categorized into two groups: the peptides belonging to the first group are active against both microbial and cancer cells but are ineffective towards healthy cells; the second group of ACPs includes peptides that are cytotoxic in a non-specific way (active on microbial cells, and healthy and tumoral mammalian cells) [128]. ACPs, like AMPs, can destroy the cells by membrane lysis or pore formation or can implement different routes, such as the disruption or penetration of the mitochondrial and/or nuclear membranes, finally inducing apoptosis. It has been speculated that ACPs can be more selective toward cancer cells not only for the anionic characteristics of cell membranes but also for their different lipid and cholesterol content with respect to normal cells that favor peptide penetration [128].

The fight against cancer also includes the regulation of biological pathways occurring at the cellular level and governing processes such as cell migration or angiogenesis, and in this respect, peptides can also function as interactors and regulators of protein targets with pro-oncogenic functions [130]. Moreover, peptides can be conjugated to anticancer molecules, which cannot penetrate the cells, and be employed as carriers for drug delivery [129]. Although peptides display several convenient characteristics for anticancer therapy, they are susceptible to proteolytic degradation (See Section 1.2). Nevertheless, this aspect can be improved with different modifications such as changes in the backbone chemistry, the incorporation of non-natural amino acids, and cyclization, as described in previous sections [126]. The growing interest in the development of ACPs has led to the creation of specific databases [131] or chemoinformatic and bioinformatic tools devoted to this research field [126,131,132]. In the next section, a few case studies related to the use of molecular modeling-based virtual screening strategies to find ACPs will be reported.

#### 3.2.2. Case Studies Related to Cancer Research

**Figure 1:**
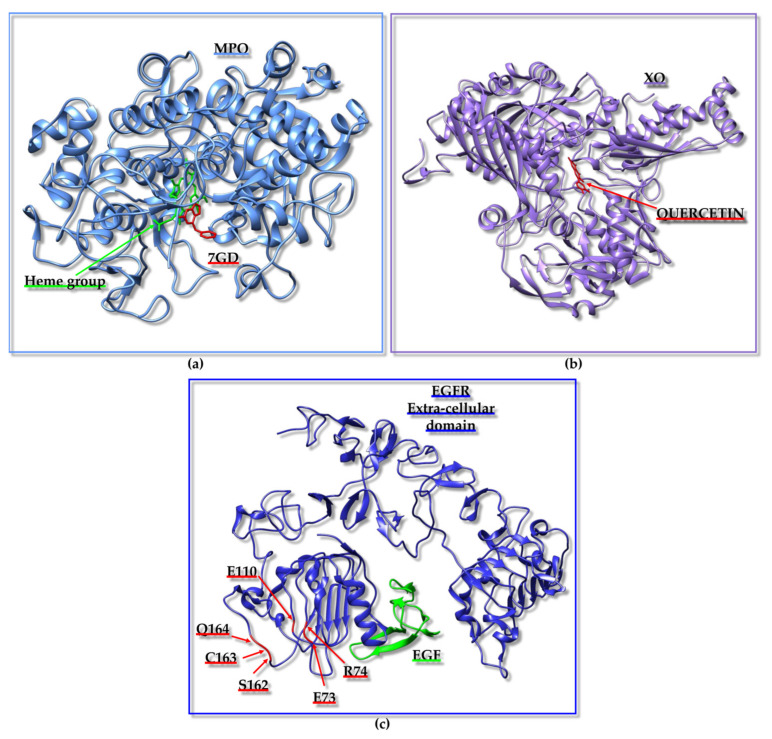
Examples of protein targets used in virtual screening approaches to find ACPs. (**a,b**) Identification of ACPs from plant seeds. (**a**) MPO (turquoise) in complex with the 7GD compound (red) and the heme group (green) (crystal structure with PDB ID: 6WYD [136], chains A and B); (**b**) XO (violet) in complex with quercetin (red) (crystal structure with PDB ID: 3NVY [137], chain C). (**c**) The structure of EGFR extracellular domain (blue) in complex with EGF (green) (crystal structure with PDB ID: 1NQL [147], chains A and B). The backbone of protein residues selected for the design of a virtual peptide library are colored in red, labelled with the one-letter amino acid codes and sequence numbers, and further indicated with arrows.

Peptides from plants have several bioactive potentials, as they can show antioxidant, antihypertensive, antimicrobial, and antitumor activities. A computational study explored the possible anticancer properties of a set of antioxidant peptides from plant seeds [133]. The perturbation of ROS (Reactive Oxygen Species) homeostasis is associated with cancer, and antioxidant peptides may intervene in its regulation. The regulation of ROS homeostasis is governed by enzymes with oxidative and antioxidative activities, such as the heme peroxidase MPO (Myeloperoxidase), XO (Xanthine oxidase), and NADPH (Nicotinamide Adenine Dinucleotide Phosphate) oxidase, and by the modulation of antioxidant gene expression. NADPH oxidase is highly expressed in different types of cancer, and its activity is supported by the interaction between p47phox (phox:phagocyte oxidase) and p22phox proteins. Thus, the inhibition of the p47phox/p22phox complex may be explored in anticancer drug discovery. Nrf2 (Nuclear factor erythroid 2-related factor 2) is a transcription factor implied in the activation of genes involved in protection against oxidative stress in cancer cells and its degradation is triggered by interaction with Keap1 (Kelch-like ECH-associated protein 1). Molecules able to hamper the formation of the Nrf2/Keap1 complex could work as anticancer agents by preventing Nrf2 degradation. Due to their importance in ROS homeostasis, MPO, XO, Keap1, and p47phox were chosen as protein targets for the identification of new ACPs. A virtual library of 667 peptides from legumes, cereals, and the seeds of plants, such as oil palm and coconut, with different antioxidant activities was assembled following a detailed peptide search through the literature and the PlantPepDB database [134]. Among the library components, 592 peptides, with a length between 4 and 50 residues and lacking non-natural amino acids, were analyzed with the AntiCP 2.0 tool [135] for anticancer potential, non-toxicity, non-allergenicity, non-hemolyticity. At the end of this screening, five candidates were selected for molecular docking experiments to check their ability to interact with target proteins. The structures of MPO in complex with the compound “7GD” (7-benzyl-1H-[1,2,3]triazolo[4,5-b]pyridin-5-amine) (PDB ID: 6WYD [136]) (Figure 1a), XO in complex with quercetin (PDB ID: 3NVY [137]) (Figure 1b), Keap1 in complex with an Nrf2 peptide (PDB ID: 2FLU [138]) and p47phox in complex with a p22phox peptide (PDB ID: 1WLP [139]) were retrieved from the PDB and the regions of interaction with their ligands were used as binding pockets during docking calculations (Figure 1a,b and Table 1). Peptide 3D structures were built with PEP-FOLD3 [140], and docking runs were performed with Autodock Vina [72] through Webina 1.0.3 [141] and HPEPDOCK [71]. Among the well-predicted peptide hits, “LYSPH” (Table 1), a peptide from cherry seeds, was found to be the most promising one in binding MPO, XO, and Keap1. Instead, p47phox was best targeted in silico by the “PSYLNTPLL” peptide (Table 1) from tomato seeds. The peptides used in the molecular docking were also tested with different computational tools to predict their potential to function as anticancer therapeutics: MLCCP (machine-learning-based prediction of cell-penetrating peptides) [142], B3Pred (blood–brain barrier penetrating peptides) [143], PlifePred (predicting peptide half-life) [144] and BIOPEP-UWM (Bioactive peptides database—University of Warmia and Mazury) [145]. Among the five tested peptides, “LYSPH” and “PSYLNTPLL” were predicted to be able to penetrate cells and the blood–brain barrier but to be susceptible to GI digestion and to have a plasma half-life lower than other anticancer peptides. Computational alanine scanning mutagenesis performed with BUDE (Bristol University Docking Engine) Alanine Scan [146] suggested that in the complexes made up of MPO, XO, Keap1, and p47phox and selected peptides, Tyr residues play important roles. For example, mutations of Tyr in Ala in “LYSPH” and “PSYLNTPLL” strongly increased the free energies of binding (ΔΔG) associated with the peptides in complex with MPO/XO/Keap1, and with p47phox, respectively. Finally, molecular dynamic simulations were run with GROMACS 2020 [87] to analyze the dynamic stability of each selected peptide against MPO, XO, Keap1, and p47phox proteins. In conclusion, the computational work speculated that “LYSPH” could represent a peptide able to block the activity of MPO, and XO enzymes and/or interfere with Keap1–Nrf2 complex formation, whereas the “PSYLNTPLL” peptide was suggested to function as a potential p47phox ligand which could inhibit the interaction of this protein with p22phox and the consequent activation of NADPH oxidase [133]. The real ability of these peptides to interact with target proteins and modulate their cancer-promoting mechanism remains to be unveiled by means of experimental approaches.

The Epidermal growth factor receptor (EGFR) is a tyrosine kinase receptor expressed on the membranes of epithelial cells. EGFR is activated by different endogenous ligands, such as Epidermal Growth Factor (EGF), and regulates physiological cellular functions. This receptor is overexpressed in several human cancers, including lung, breast, bladder, and ovarian cancers, where it is associated with the growth and progression of tumors, regulating the angiogenesis, invasion, and metastasis of cancer cells. EGFR was the target of a study in which a peptide ligand (D4: LARLLT) was identified starting from a computational approach (Table 1) [149]. First, the X-ray structure of the extracellular region of EGFR was retrieved from PDB (PDB ID: 1NQL [147]), and then, an analysis was conducted with the PSCAN program to find a binding pocket to be targeted by peptide ligands. To this aim, a group of six amino acids surrounding the identified EGFR binding pocket (Q164, C163, S162, E110, E73, and R74) (Figure 1) was implemented to design a library of peptides using the theory of sense and antisense peptide interaction, based on the concept that peptide sequences originating by complementary nucleic acid portions are more prone to specifically make interactions. This method allowed for the design of 132 6-mer peptides that were all docked against the EGFR binding pocket (Figure 1c) with AutoDock3 [148]. Considering the docking energy values, 20 peptides were selected, and 10 of them were synthesized. Finally, the D4 peptide was considered the best choice for further in vitro and in vivo experiments. The peptide was first conjugated with PEG (polyethylene glycol) to insert it into a liposome membrane. The liposomes conjugated with D4 peptide were labelled with the fluorescent probe rhodamine and tested on H1299 cancer cells over-expressing EGFR. These experiments demonstrated that the D4-conjugated liposomes bind and penetrate the H1299 cells by endocytosis. In in vivo fluorescence experiments in mice bearing an H1299 tumor, the peptide accumulated at cancer sites within the mice body, with a circulating half-life of approximately 6 h. In vitro and in vivo experiments were also performed using liposomes conjugated with a scrambled D4 peptide as a negative control that did not show the same D4 specificity to penetrate cancer cells and accumulate in tumor mice regions. In the end, the computational approach led to the identification of the D4 peptide that could be further employed to develop improved EGFR ligands to be conjugated to delivery systems for cancer treatment [149].

EGFR was the target of another preliminary computational study in which a few dipeptides conjugated with the compound Ebselen were designed [153] (Table 1). Ebselen is a selenium-containing compound with anti-inflammatory, antioxidant, and cytoprotective activities that has been reported to possess anticancer potential in different tumor types. Ebselen derivatives also present anti-tumoral functions as they have cytostatic and cytotoxic capabilities. The PDB file of the EGFR kinase domain in complex with a small molecule (PDB ID: 3W2S [150]) was used as a target to perform docking studies with three Ebselen dipeptide derivatives (Eb-WD, Eb-WE, Eb-WK). Possible protein binding pockets were searched with the CASTp (Computed Atlas of Surface Topography of proteins) software (v. 3.0) [151], and the 3D structures of the Ebselen-conjugated peptides were obtained with the Chemsketch software from ACD labs [152]. The docking studies to analyze the interaction between EGFR and different Eb derivatives were carried out with AutoDockVina [72], and the resulting docking energies were compared with those obtained for the EGFR–doxorubicin complex. Among the screened molecules, Eb-WE (Table 1) showed an interaction energy to EGFR close to that obtained for the EGFR–doxorubicin complex. Thus, this Ebselen derivative may have anticancer potential, but of course, experimental validation is needed to confirm these computational data [153].

Computational techniques have been used to design peptide inhibitors of the T-cell CTLA4 (Cytotoxic T-Lymphocyte–Associated antigen 4) receptor [162]. CTL4 is one of the receptors expressed on immune cells and is involved in the immune self-tolerance pathways that are targeted in cancer immunotherapy. Indeed, immune cells can intervene in the tumor microenvironment by recognizing and destroying antigens specific to cancer cells. However, cancer cells can evade these self-tolerance mechanisms by involving inhibitory receptors expressed on immune cells, such as CTL4, that, together with their ligands, are targeted by immunotherapy. Peptides might work better as inhibitors of these receptors with respect to antibodies that are more expensive and difficult to store. The X-ray structure of the CTLA4 receptor, extracted from the complex with the B7-2 protein (PDB ID: 1I85 [154]) (Table 1), was used as a template to generate peptide sequences by means of in silico techniques (including flexible docking and MD simulations). First, the residues mainly involved in the CTLA4/B7-2 complex formation were predicted using the KFC (Knowledge-based FADE and Contacts) web server [155] and, consequently, a B7-2 region to use as a peptide template was identified. The structure of the template peptide was predicted with the Phyre (Protein Homology/analogY Recognition Engine) 2 web server [156] and used in flexible docking runs against CTLA4 through Rosetta FlexPepDock [157]. A few of the protein–peptide models generated during this step were selected and structurally optimized with PyRosetta [158]. Peptide sequences were then head-to-tail cyclized to improve their resistance to protease degradation, and their stabilities in the CTLA4-bound forms were predicted using MD simulations by the NAMD 2.13 program [163] and MM-GBSA binding-free energy calculations [160]. The best-performing protein–peptide complexes were further submitted to an MD free energy calculation in explicit solvent by using the BFEE (Binding Free Energy Estimator) module of VMD (Visual Molecular Dynamics) [161], and the resulting best models were further implemented in flexible docking to achieve an additional optimization cycle.

The peptides were also experimentally tested by means of bio-layer interferometry to confirm their ability to bind CTLA4 and by in vitro and in vivo experiments in Lewis lung carcinoma (LLC) cells and in orthotropic Lewis lung carcinoma allograft models to verify their capacity to block tumor cell growth. One of the peptides, the cyclic EIDTVLTPTGWVAKRYS, was identified as the most promising immunomodulator in cancer therapy [162].

Another protein involved in the inhibition of the immune response against cancer cells, HVEM (herpesvirus entry mediator), was the target of a study in which linear and cyclic peptides were designed as antagonists of its interaction with BTLA (B- and T-lymphocyte attenuator). Experimental studies and computational techniques were nicely combined within this study. For peptide design, the N-terminal fragment of the gD (glycoprotein D) protein, that binds HVEM and allows for the starting of infections by herpes simplex virus-1 and -2 through a variety of entry mechanisms, was employed. Structure-based drug design and MM-GBSA analysis were implemented as computational tools to select lead compounds to be experimentally validated through diverse experimental analyses [including ELISA (Enzyme-Linked Immunosorbent Assay) and cellular-based reporter assays]. For the best peptide candidates, docking simulations were also performed to predict the protein–peptide binding topology. One of the peptides, provided with a cyclic arrangement and a disulfide bridge (i.e., gD(1–36)(K10C-T29C)), was considered the best candidate to block BTLA inhibitory function and increase the immune response towards cancer cells [164].

In our laboratories, we have long been studying the tyrosine kinase receptor EphA2 and employing computational approaches to identify peptide inhibitors of its interaction with the lipid phosphatase Ship2 (SH2 domain-containing inositol phosphate 5-phosphatase 2) [124]. The EphA2 receptor is overexpressed in several cancer cell types, with recognized roles in cancer onset and progression. The EphA2 cytosolic Sam domain binds to the Sam domain of the lipid phosphatase Ship2, and this interaction is responsible for the inhibition of EphA2 receptor endocytosis and degradation and is mainly linked to pro-oncogenic outcomes. Different computational-based drug design approaches were implemented to develop virtual peptide libraries focused on Sam domains that were subsequently employed in docking-based virtual screenings to identify peptide inhibitors of the EphA2-Sam/Ship2-Sam complex with possible anticancer activity [122,123,124].

ACPs can also be identified from virtual peptide libraries with computational techniques not including molecular docking. For instance, a peptide from the milk protein alpha-lactalbumin with cytotoxic activity on A549 lung cancer cells was identified using machine learning methods as a virtual screening approach to identify peptides with relevant physicochemical and anticancer properties [165].

### 3.3. Antimicrobial/Antiviral Peptides

Antimicrobial resistance (AMR) is a serious warning to worldwide health. The excessive use of antibiotics in farms as an addition to animal feed and in humans to treat bacterial infections are among the key factors contributing to the diffusion of AMR, which mainly affects clinical fields related to surgery, transplantation, and the treatment of inflammations [166]. Viral agents also can develop AMR to vaccines and antiviral therapeutics, and this concern is particularly worrying if associated with the pandemic risk arising from some viral pathogens [167]. The most recent COVID-19 pandemic is a case in which AMR has been among the highest priorities for public health.

These issues point out the necessity for drug discovery campaigns to find new therapeutics able to overcome AMR. Antimicrobial peptides (AMPs), with the ability to perturb pathogen membranes or to regulate pathogen or host protein biological pathways, could be a good source of new antibacterial and antiviral therapeutics.

#### 3.3.1. Introduction

AMPs can be found in all living organisms as they are part of the host defense machinery against pathogens. AMPs can directly or indirectly kill pathogen cells, by modulating immunity pathways in response to pathogen entry into host cells [168]. The biological activities of AMPs depend on their physiochemical properties, such as the net charge, the conformation, and hydrophobicity. These peptides are usually positively charged, with a length between 10 and 50 amino acids, and characterized by a variety of conformations (α-helical, β-sheet, or linear without specific secondary structure elements) [169]. Different models to explain the mechanisms that AMPs employ to disrupt cell membranes have been proposed, such as the barrel-stave, the carpet-like, the toroidal pore, and the detergent-like models [168]. AMPs are generally considered in the context of anti-bacterial treatments, but the Antimicrobial Peptide Database (APD) [170] contains 3569 antimicrobial peptides from six life kingdoms (https://aps.unmc.edu/, accessed on 15 November 2023) and 207 of them are classified as antiviral peptides. Viral AMPs can act as well by different mechanisms, such as the inhibition of viral infection by preventing interaction with host cells or by inhibiting the transcription and translation processes of the viral genes [168]. As for ACPs, databases [170,171] and specific computational tools, including machine learning methods [171,172,173,174], have been developed to help the scientific community in the drug discovery field and to understand the mechanisms of action of AMPs.

The next paragraphs will focus on several studies related to the search using virtual screening and additional in silico tools of antiviral peptide agents against SARS-CoV-2.

#### 3.3.2. Case Studies: Targeting SARS-CoV-2

The severe acute respiratory syndrome coronavirus 2 (SARS-CoV-2) has been responsible for 6.985.964 deaths worldwide since the onset of the coronavirus disease 2019 (COVID-19) pandemic till the beginning of December 2023, thus justifying the many efforts made for the continuous development of novel treatments against this disease [175]. SARS-CoV-2, its mechanism of action, and many computational tools available for the development of small molecule ligands against viral proteins have already been described in several previous reviews [176,177,178,179,180,181,182]. Briefly, SARS-CoV-2 belongs to the enveloped viruses, including a positive-sense single-stranded RNA, and its genome encodes different viral proteins supporting its infective action [176]. For instance, the SARS-CoV-2 spike protein binds to the human receptor angiotensin-converting enzyme 2 (ACE2) through a receptor-binding domain (RBD), thus allowing viral entry into host cells [176,183,184,185,186,187,188,189,190]. In addition, the 3C-like protease (3CLpro), also known as main protease (Mpro), is a viral protein with a key role in the processing of the polyprotein precursors pp1a and pp1b into the functional proteins necessary for viral propagation [176,191,192,193,194].

The search for the peptide inhibitors of SARS-CoV-2 proteins through in silico approaches can be achieved following diverse canonical key stages and combining docking studies and molecular dynamics simulations [183].

**Figure 2:**
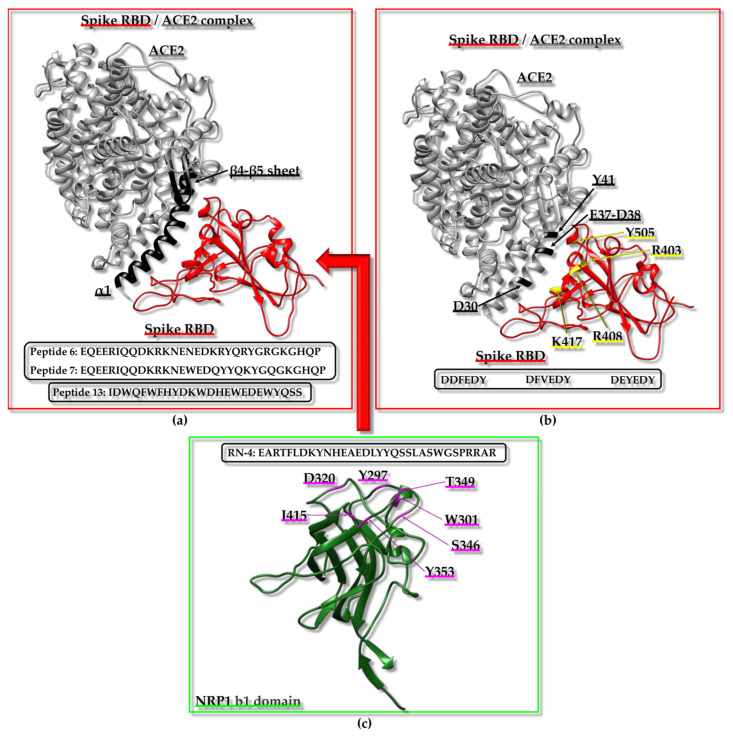
In silico approaches to discover antiviral peptides targeting the receptor-binding domain of SARS-CoV-2 spike protein (spike RBD) (**a,b**) and neuropilin 1 (NRP1) receptor (**c**). (**a**) The crystal structure of spike RBD in complex with ACE2 receptor peptidase domain (PD) (PDB ID: 6M0J [196]) is shown in a ribbon representation where the spike RBD is colored in red and ACE2 in grey with regions important for the interaction (i.e., α1 helix and β4–β5 sheets) highlighted in black. Peptides 6, 7, and 13, the sequences of which are reported in the one-letter amino acid code, were identified through in silico binding studies of the spike RBD structure extracted from the shown PDB entry [184,187]. (**b**) X-ray structure of spike RBD (red)/ACE2 (grey) complex (PDB ID: 6LZG [202]). Hot spot residues on ACE and spike RBD are colored in black and yellow, respectively. The sequences of a few RBD spike-targeting peptides discovered by docking-based virtual screening are shown [185]. (**c**) Crystal structure of NRP1 b1 domain (green) (PDB ID: 7JJC, chain A extracted from the complex with the CendR peptide [204]). Residues of the NRP1 peptide binding pocket are labeled and colored in magenta. The RNF4 peptide is an in silico-identified dual ligand of spike RDB and NRP1 b1 domain [186].

Several in silico studies concerning the inhibition of viral entry into host cells by blocking the interaction of the RBD domain of the SARS-CoV-2 spike protein (spike RBD) with the human ACE2 receptor have been reported in the literature (Figure 2a) [183,184,185,187,195]. In one such study, the authors retrieved from the PDB several structures of the RBD from the SARS-CoV-2 spike glycoprotein/ACE2 complex (i.e., PDB ID: 6M0J [196], 7C8D [197], and 7A95 [198]) and deeply analyzed them also through the MM-GBSA approach. MM-GBSA was used to evaluate the per-residue contribution to the binding free energy and predict the crucial amino acids at the binding interface, also accounting for the reproducibility of results in all the considered structures [195]. This investigation revealed that the interacting residues were mainly positioned within two ACE2 regions (i.e., the α1 helix and the β4-β5 sheets, Figure 2a). Next, the gained information was employed to design a 49-mer peptide (SARS-CoV-2 PEP 49) encompassing the amino acids of both the α1 and β4-β5 regions of ACE2, the 3D structure of which was predicted with PEP-FOLD3 [140] and implemented in docking studies against the SARS-CoV-2 spike protein. The docking results pointed out that the designed peptide could target the RBD domain of the spike protein with a better interaction energy than ACE2 itself. In the end, the in silico data showed that PEP49 could potentially work as a good inhibitor of viral entry and consequently be implemented to design original antiviral therapeutics [195].

Nevertheless, a strategy called EvoDesign was employed to generate in silico several peptides targeting the spike RBD to block viral entry into host cells [199]. Starting from the analyses of a crystal structure of the SARS-CoV-2 spike RBD/ACE2 complex (PDB ID: 6M0J [196]), a peptide scaffold was first built by combining, through a glycine linker, two segments extracted from the ACE2 receptor interface. This initial scaffold was used as a starting point in bioinformatic tools for de novo protein design (i.e., EvoEF2 [200] and EvoDesign [201]) to generate novel peptide sequences optimized for interaction affinity towards the SARS-CoV-2 spike RBD. Twelve of the best peptides, according to the in silico binding studies, were also subjected to another computational screening. In this secondary screening, MD simulations of the spike RBD/peptide complexes were carried out to analyze the binding affinities and stability, looking at diverse features like the hydrogen bonding aptitude and RMSD (Root Mean Square Deviation) values. The results, combined with the predictions of the peptide secondary structures and stabilities in aqueous solution, led in the end to the choice of the best peptide candidates (e.g., Peptide 6 and Peptide 7 in Figure 2a) that could be conjugated to a graphene sheet or a carbon nanotube to generate a bio-sensor for SARS-CoV-2 detection [184].

In a similar computational approach, the binding of the SARS-CoV-2 spike RBD to ACE2 (PDB ID: 6LZG [202]) was investigated through MD and MM-PBSA free energy calculations to establish the key residues at the binding interface of the two proteins (Figure 2b). Based on the hot spot residues identified on ACE2 (i.e., D30, E37, D38, and Y41), a library of 6-mer peptides (*consensus* sequence: “DX1X2EDY” where X1 and X2 represent any among the 20 natural amino acids) was designed. Through docking-based virtual screening against the spike RBD, six peptides were selected and further validated by MD. Finally, three peptides (DDFEDY, DEYEDY, and DFVEDY in Figure 2b) resulted as the best candidate inhibitors of viral entry and were further analyzed by bioinformatic tools to estimate the allergenicity, toxicity, and solubility [185].

An additional in silico approach to attack viral infectivity by blocking the entry stage started from a diverse virtual library of peptides, but again exploited the concept that a peptide somehow similar to the ACE2 sequence responsible for interaction with the spike protein might work as an inhibitor of the spike RBD/ACE2 complex and thus block viral infection. In this context, a peptide library was assembled by mutating ACE2 residues not involved in binding to spike RBD according to the analyses of experimental structures. In detail, starting from a peptide fragment encompassing the α1 helix in ACE2, mutations were inserted at 12 non-interacting amino acid sites; the introduction of point and multiple mutations led to the generation of 136 peptides. Docking-based virtual screening against the SARS-CoV-2 spike RBD (Figure 2a) was conducted, and seven peptides with potential high binding affinities could be predicted. The MD simulations further pointed out one potential peptide inhibitor (peptide 13 in Figure 2a) of viral entry with a~ 3-fold increased interaction affinity for spike RBD with respect to a reference ACE2 peptide encompassing the α1 helix [187].

Although these studies [185,187,195] provide useful insights for the design of potential peptide inhibitors of viral infectivity modulating the viral entry stage, the lack of experimental validation remains a major drawback. On the contrary, an elegant study by Hu and collaborators reported on the successful in silico identification and experimental validation of a peptide able to reduce SARS-CoV-2 entry into host cells by targeting simultaneously the RBD of the SARS-CoV-2 spike protein and human neuropilin-1 (NRP1) [186]. The NRP1 receptor represents another important player in SARS-CoV-2 entry as it has been proposed as a co-receptor that might adjuvate ACE2 to favor virus attachment to olfactory and respiratory epithelial cells, thus enhancing infectivity [186,203]. The NRP1 extracellular b1 domain is generally used as the target to find NRP1 inhibitors; furin is a host protease that cleaves the SARS-CoV-2 precursor spike S protein into the S1 and S2 fragments and consequently generates a basic stretch at the C-terminal end of S1 (i.e., the C-end rule (CendR) motif) responsible for interacting with the NRP1 receptor [204]. To identify peptides able to bind the spike RBD and NRP1 b1 domain simultaneously, virtual screening was conducted by employing both pharmacophore-based docking and structure-based docking. The software Molecular Operating Environment (MOE [205], Chemical Computing Group Inc, Montreal, Quebec, Canada) was used for the pharmacophore modeling. A library composed of 24,000 peptides was built by the QuaSAR-CombiGen module of MOE through the random creation of connections between fragments of different lengths (i.e., 4-mer, 7-mer, 9-mer, 12-mer) [186]. The resulting 2D peptide models were converted into 3D structures; meanwhile, the crystal structure of ACE2 in complex with the SARS-CoV-2 spike RBD (PDB ID: 6M0J [196]) and the crystal structure of the human NRP1 b1 domain in complex with the SARS-CoV-2 S1 CendR peptide (PDB ID: 7JJC [204]) (Figure 2a,c) were downloaded from PDB, hydrogens were added, and the protonation state optimized. Next, the MOE program was employed to deeply analyze the interactions between the SARS-CoV-2 spike RBD and ACE2 from the crystal structure [186]. The resulting data were exploited by the Pharmacophore Editor module of MOE to provide the pharmacophore profile for spike RBD (i.e., an ensemble of characteristics that peptides should have to match the features of spike RBD binding pocket). A pharmacophore-based docking simulation was conducted to identify potential peptide ligands of spike RBD through the docking module of MOE. In detail, to screen the peptide database through pharmacophore-based docking, the pharmacophore model was used as a 3D query, while the binding site included hot spot residues on the interaction surface of spike RDB. The binding free energies for the spike RBD/peptide complexes were evaluated based on the docking scores. The peptides provided with docking scores lower than a chosen cut-off (i.e., −13.5 kcal/mol) were further assayed through structure-based docking into the NRP1 b1 active site, and the top five peptides were selected for in vitro biological testing. This clever approach was conducted for the identification of one peptide (i.e., RN-4, Figure 2c) able to simultaneously bind both the spike RBD and NRP1 b1. In in vitro tests, RN-4 showed nanomolar affinities against both proteins, and in the pseudovirus infection assay, the capacity to significantly lower the SARS-CoV-2 entry into cells without provoking substantial adverse effects was also demonstrated [186].

To discover peptide ligands of the spike RBD that could reduce SARS-CoV-2 infectivity by blocking viral entry, a work by Ramirez-Acosta and collaborators reported on a virtual screening strategy employing antimicrobial peptides [190]. A library of 104 peptides was assembled starting from the Antimicrobial Peptide Database (APD) of the University of Nebraska Medical Center [170,206] by focusing on those peptides with known antiviral activities [190]. Since lysozyme is an innate immune system component with antimicrobial activity, three lysozyme fragments were inserted into the library [190]. In addition, for comparison purposes, an ACE2-derived peptide (i.e., “IEEQAKTFLDKFNHEAEDLFYQSS”) was included in the screening dataset. The structures of the preselected peptides that could not be retrieved from the PDB were predicted by different sequence-based in silico tools (I-TASSER (Iterative Threading ASSEmbly Refinement) [207,208] and PEP-FOLD 3.5 [140]) and then used as input for a docking-based virtual screening against the spike RBD [190]. The docking analysis predicted for the twelve antimicrobial peptides and the three lysozyme-derived peptides better binding affinities for the spike RBD than that predicted for the ACE2 reference peptide. Thus, after analysis of the most recurrent amino acid types interacting with the RBD and based on H-bond pattern, extra optimized peptides were designed and employed in docking studies against the spike RBD. The ability of the peptides to escape the immune system was also investigated computationally through the TepiTool, and for a few of them, a low immunogenic response was predicted [209]. In the end, a few optimized peptides with enhanced predicted binding affinity towards diverse RBD variants (including the Delta one and a theoretic form derived by combining the Alpha, Beta, and Gamma variants) were identified [190]. This represents a promising protocol for discovering antiviral agents.

Thakkar and colleagues reported instead on the development, through the combination of protein design programs and molecular dynamic simulations, of a 17-mer stapled peptide (pep39) targeting the spike RBD and blocking its interaction with human ACE2 [210]. The initial design started from a template peptide encompassing residues 26–42 of the ACE2 α1-helix and from the structure of the human ACE2/spike RBD complex (PDB ID: 6LZG [202]) (Figure 2b). The structure-based FlexPepDock protocol [157] was implemented to build a peptide that is able to interact with the spike RBD. Following this strategy, a wide search of the conformational space accessible to the peptide backbone was achieved using FlexPepDock. Next, docking poses provided with both low energies and a certain degree of structural similarity with the template peptide were selected. To enhance the affinity of the chosen poses for the spike RBD, the protocol proceeded by substitutions with the common 20 amino acids and the optimization of the sidechains and rotamers. Several optimization cycles led to many diverse possible sequences, but certain amino acid residues appeared to be preferred in specific peptide positions: proline and glycine at positions 8 and 10, respectively, and hydrophobic amino acids at positions 2, 12, 13, and 14. The first 41 optimized peptide structures in complex with the spike RBD were subjected to molecular dynamics simulations. The MM-GBSA binding free energy calculations allowed for the further filtering of 9/41 optimized peptides. Moreover, to favor structure resemblance with the ACE2 α1 helix, a propene staple and an amide bond between the side chains of a negatively charged residue and a positively charged one were introduced into ad hoc chosen positions to enhance the peptide helical structures. The MD simulations coupled to the MM-GBSA binding free energy calculations demonstrated the largest stabilities of the stapled peptide conformations, as well as their higher ability to remain in the bound state. The analysis of the MD trajectories demonstrated that the so-called “pep39” peptide outperformed and was able to interact well with the spike RBD in the ACE2 binding site. This most promising “pep39” peptide was experimentally validated. The interaction studies through bio-layer interferometry demonstrated the binding of pep39 to the spike RBD (dissociation constant K_D_ = 570 ± 50 nM) and its delta variant (K_D_ = 4.1 ± 1.4 μM). In addition, the cell-based assays suggested that this stapled peptide was able to block SARS-CoV-2 replication and could function as a potential anti-COVID-19 therapeutic agent [210].

**Figure 3:**
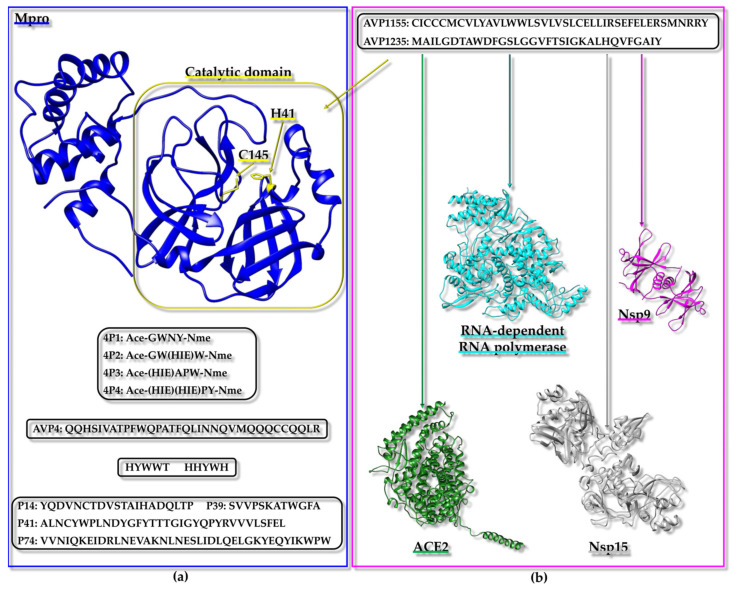
(**a**) The ribbon representation of free Mpro (crystal structure with PDB ID: 6Y2E [212]) is reported in blue, the catalytic domain is highlighted with a yellow box, and the side chains of the residues (Cys145 and His41), forming the catalytic dyad, are shown. The amino acid sequences of several in silico identified potential peptide inhibitors of Mpro are indicated [189,191,192,214]. The “4P” peptide series contains only amino acids in D-configuration; Ace stands for the N-terminal Acetyl group, Nme stands for N-methyl amidation at C-termini, and HIE represents the E tautomer of histidine [189]. (**b**) Electron microscopy (EM) structure of full-length ACE2 receptor (green) (extracted from the RBD/ACE2-B0AT1 complex PDB ID: 6M17 [215], chain D) and diverse SARS-CoV-2 proteins: RNA-dependent RNA polymerase (cyan) (extracted from the EM structure of SARS-CoV-2 RNA-dependent RNA polymerase in complex with cofactors with PDB ID: 6M71 [216], chain A); Nsp15 (grey) (crystal structure—dimeric form—PDB ID: 6VWW [217], chains A and B); Nsp9 (magenta) (crystal structure -dimeric form- PDB ID: 6W4B [218], chains A and B). Antiviral peptides AVP1155 and AVP1235 [219] were identified through a multi-target in silico screening against all proteins in panel (**b**) as well as Mpro (**a**).

A diverse computational strategy relying on the combination of docking analysis and MD simulations provided four dual peptide inhibitors targeting both the spike RBD and the Mpro protease (Figure 3a). The screening of a peptide library was achieved through the docking module in the MOE [205] software. The crystal structures of both the viral protease Mpro complexed with a cyclic peptide (PDB ID: 7RNW [211]) and spike RBD in complex with ACE2 (PDB ID: 6M0J [196]) were implemented in this study. A virtual peptide library was combinatorially built using the QuaSAR-CombiGen module of MOE starting from diverse peptide fragments (i.e., 15-mer cyclic peptides, heptapeptides, and 16- and 18-mer linear peptides) and included 27,000 cyclic peptides made up of an α-helix and a cyclic segment [188]. The α-helix was inserted considering that this structure element in ACE is important for the interaction with the spike RBD (Figure 2). All the peptides were first screened against Mpro, and the best hits (i.e., docking scores lower than −13.7 kcal/mol) were docked towards the spike RBD. The best four dual ligands were further validated using MD simulations to analyze the stabilities of the protein–peptide complexes. Finally, the four in silico hits were experimentally validated. First, the MST (Microscale thermophoresis) interaction assays provided evidence of peptide binding to both the Mpro and spike RBD with dissociation constants in the nanomolar range and an increase in the binding affinity compared to two other peptides used as positive controls [188]. In addition, a pseudovirus infection assay showed that more than half of the SARS-CoV-2 pseudovirus was inhibited by these peptides without considerable cytotoxicity to the host cells [188].

As mentioned before, the protease Mpro represents another appealing target in antiviral drug discovery to counteract SARS-CoV-2 infection. To target Mpro, a virtual peptide library was designed considering that D-amino acids provide some advantages when incorporated into a peptide sequence (i.e., a higher resistance to proteases, improved intestinal absorption upon oral administration, and low or absent immunogenicity), and that small peptides (i.e., 3-mer and 4-mer) perform better in docking algorithms since they possess less freely-rotatable bonds [189]. A virtual screening was conducted against the crystallographic structure of free Mpro (PDB ID: 6Y2E [212]) (Figure 3a) [189]. A library of D-tri- and tetra-peptides was built through specific commands of Amber20 [213]; the peptides were assembled by considering combinations of the common 21 residues (i.e., the 20 natural amino acids, and considering both neutral tautomers of histidine), and including the N-terminal acetyl and C-terminal N-methyl amide capping groups. Starting from the natural amino acids, the L-configuration was inverted to the D-configuration for each residue, but for Ile and Thr, modeling was performed to obtain D-allo-isoleucine and D-allo-threonine diastereomers. The 3D structures of each peptide were generated, and were energy minimized [189]. Then, structure-based virtual screening was first conducted by docking the D-peptide database in the Mpro active site; the resulting predicted Mpro/peptide complexes were subjected to rescoring through the MM-GBSA approach and the evaluation of binding free energies, followed by MD simulations and the analysis of different parameters [i.e., RMSD, RMSF (root mean square fluctuation), and the number of H-bonds] [189]. The proposed protocol provided four D-tetrapeptides (i.e., 4P1, 4P2, 4P3, and 4P4 in Figure 3a) that were evaluated in in vitro enzymatic assays and demonstrated the capacity to block from 50 to 85% of the Mpro activity, thus proving that the implemented computational strategy could successfully be conducted to identify novel anti SARS-CoV-2 agents functioning as inhibitors of the Mpro protease [189].

In order to find Mpro inhibitors, another study aimed to generate a peptide library by the in silico digestion of the four more abundant proteins in rice bran (i.e., albumin, glutelin, globulin, and prolamin) by the proteases pepsin, trypsin, and chymotrypsin [191]. Then, the results were analyzed by diverse bioinformatic tools to predict potential antiviral/antimicrobial peptides (several programs were implemented: AVPpred [220], Meta-iAVP [221], AMPfun [222], and ENNAVIA (Employing Neural Networks for Antiviral Activity) [223]). The peptide amino acid composition and cytotoxicity were also estimated by the COPid (COmposition based Protein Identification) [224] and ToxinPred [225] tools. Through all these analyses, 10 antiviral peptides were chosen, and their 3D structures were predicted by PEP-FOLD 3 [140] and used as the input for docking analyses conducted through the GalaxyPepDock [70] program against the crystal structure of Mpro (PDB ID: 7C2Q [226]) [191]. This stage indicated that all peptides target a region of Mpro near its active site, and the best ligand, according to the docking scores, was the AVP4 peptide (Figure 3a). The Mpro/AVP4 peptide complex was further analyzed by MD simulations that pointed out a prolonged and robust interaction, thus confirming the validity of this virtual screening approach [191].

As mentioned in Section 2.1, genetic algorithms can be employed to generate peptide libraries and lower the high computing power usually requested to handle libraries made up of tens of thousands of components [192]. In a computational strategy to find Mpro inhibitors, the Mpro structure (PDB ID: 6LZE [227]) was employed to discover high-affinity peptide ligands by means of a genetic algorithm. A group of peptide sequences representing the populations was evolved through the genetic algorithm by using docking scores so that several iterations were carried out, and each time, a new population was generated from the previous one by sequence pairing, thus leading to better-predicted peptide binding affinities according to the docking scores. In other words, the implemented algorithm applied a sort of “Darwinian selection” in which the accomplishment of lower docking scores against SARS-CoV-2 Mpro was used as a criterion for evolutive steps [192]. The approach started from an initial population including 19 5-mer peptides made up by the repetition of a single residue (consensus sequence “XXXXX”, where X is the same single amino acid, chosen from the 20 natural ones, excluding proline) [192]. To achieve greater levels of sequence diversity by ensuring random pairing, the same score was attributed to each member of the starting population [192]. Starting from the second iteration, the resulting couples were subjected to a crossing-over process, during which the 50 sequence pairs with the best docking scores were saved and selected for a roulette wheel pairing step in which the new population of sequence couples is built for the next evolutive step [192]. In addition, each cycle included the removal of the worst ten sequences [192]. The protocol led to two peptides (i.e., HHYWH and HYWWT, Figure 3a) that could interact with SARS-CoV-2 Mpro with an affinity greater than those against human proteases, thus indicating the proposed strategy as a valid tool for the development of a starting lead peptide for the design of optimized potential antiviral agents [192].

As previously mentioned, an intriguing source of potential inhibitors of SARS-CoV-2 viral proteins can be supplied by the database AVPdb, which includes peptides the antiviral roles of which have been experimentally proven [194]. In addition, for the peptides included within this database, useful features are provided (like sequence, source, target virus, virus family, efficacy from the qualitative and/or quantitative point of view, assays used to establish the efficacy, the physicochemical characteristics, and PubMed references) along with a predicted structure [194]. A list of 88 antiviral peptides was extracted from AVPdb based on anti-SARS-CoV-2 activities [214], and their 3D structures were modeled by PEP-FOLD 3 [140]. These peptides were virtually screened against the X-ray structure of Mpro (PDB ID: 6LU7 [228]). The CASTp webserver [151] was employed to clearly identify the binding pocket on the surface of Mpro. Next, docking was carried out first with PatchDock [229], and then further docking runs were performed with FireDock (Fast interaction refinement in molecular docking) [230] starting from the best 10 conformations of each Mpro/peptide complex; to additionally validate the results, the docking was also conducted with ClusPro [231]. Four peptides (i.e., P14, P39, P41, and P74 in Figure 3a) scored as the best ligands, according to several docking tools, and their complexes with the Mpro active site were subjected to MD simulations to study the conformational stability [214]. Different MD-related parameters (i.e., the RMSD, solvent accessible surface area, radius of gyration, RMSF, and number of H-bonds) indicated the higher stability of the protein–peptide complexes even with respect to Mpro in the apo form [214].

In a second work [219], a set of 434 AVPs were extracted from AVPdb [194] following three main criteria: 1—the exclusion of peptides with proven activity against viruses of the coronaviridae class, as the goal was to discover original anti-SARS-CoV-2 agents, 2—a lack of cytotoxicity, and 3—a length of at least 27 residues to allow for the efficient modeling of 3D structures by the Robetta webserver [232]. The selected antiviral peptides were virtually screened by docking against both the SARS-CoV-2 Mpro (PDB ID: 6M03 [233]) and the non-structural protein 9 (Nsp9) (PDB ID: 6W4B [218]) with the ClusPro webserver [231] (Figure 3a,b). From this first screening, eleven peptides with the most promising docking scores (based also on a comparison with reference peptides) towards both targets were selected. The eleven peptides were next employed in a second docking-based virtual screening approach against other SARS-CoV-2-related targets (Figure 3b) [i.e., the ACE2 receptor-binding domain (PDB ID: 6M17 [215]); SARS-CoV-2 RNA-dependent RNA polymerase (PDB ID: 6M71 [216]), and Nsp15 endoribonuclease (PDB ID: 6VWW [217])] [219]. From the second screening, two peptides (1155 and 1235 in Figure 3b) were selected with good scores against all the proteins. In the end, the complexes of all five of the target proteins and the two best peptides were further analyzed by MD simulations to confirm the structural stability of the protein–peptide complexes [219].

In summary, during the COVID-19 pandemic, and especially during the initial outbreak, in silico approaches to fight SARS-CoV-2 flourished due to the urgent need to find therapeutic agents. Many putative peptide inhibitors of SARS-CoV-2 proteins have been identified in silico but not experimentally validated. The confirmation of the antiviral activities of these peptides could open the road to the establishment of novel therapeutics, avoiding undesired side effects.

### 3.4. Inhibiting Fibril Formation: Aβ_42_ and hIAPP as Targets

**Figure 4:**
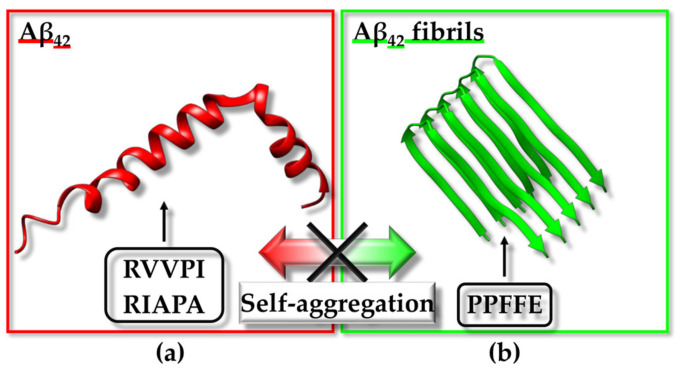
(**a**) NMR structure of Aβ_42_ monomer (red) calculated in HFIP (Hexafluoroisopropanol/water) 80/20 *v*/*v*, (PDB ID: 1IYT [237], first conformer). RVVPI and RIAPA peptides [234] that were identified by vs. against the monomeric form of Aβ_42_ could stabilize the Aβ_42_ helical conformation. (**b**) NMR structure of Aβ_42_ fibrils (green) in aqueous buffer (PDB ID: 2BEG [239], first conformer). The PPFFE peptide should work as a β-breaker and was selected by initial vs. against Aβ_42_ fibrils [235].

Virtual screening strategies have also been designed to identify peptides able to inhibit protein self-aggregation, which is often the cause of pathological events [234,235,236]. AD (Alzheimer’s disease) represents a neurodegenerative disorder due to accumulation in the extracellular space of plaques. Neuritic plaques found in the brains of AD patients are mostly composed of Aβ peptides resulting from the proteolytic cleavage of amyloid precursor proteins that form neurotoxic fibrillary β-sheet structures [237]. Aβ-(1–42) (=Aβ_42_) represents the major form found in plaques. It has been reported that monomeric Aβ_42_ might initially assume an α-helical or disordered state before switching into a β-sheet conformation. This conformational change represents the crucial stage in the Aβ fibrillogenesis process (Figure 4a,b) [238].

Starting from a previously identified inhibitor of Aβ aggregation and toxicity (i.e., the “RIIGL” peptide), a virtual peptide library was designed. In detail, this library was built by substituting diverse amino acid positions of the starting peptide with residues provided with similar side chains. For example, the first “R” in the “RIIGL” peptide was mutated in a few polar and basic amino acids (K, H) while the “I” was substituted with some non-polar aliphatic residues. The final library included 912 5-mer peptides that were virtually screened, through docking techniques, against Aβ_42_ in the monomeric form (PDB ID: 1IYT [237]) [234]. The best 10 peptide ligands, according to the docking scores, were next assessed through molecular mechanics Poisson–Boltzmann surface area (MM-PBSA) to evaluate the free energy of the interaction with Aβ_42_. Based on this analysis, the three best peptides (“RLAPV”, “RVVPI”, and “RIAPA”) (Figure 4a) were further investigated by MD simulations to study the conformational variations of Aβ_42_ in the presence and the absence of each peptide. Interestingly, the MD revealed that in the presence of the “RVVPI” and “RIAPA” peptides, there was a higher stabilization of Aβ_42_ helical states (Figure 4a) with respect to the β-sheet conformations characterizing instead the fibrillar species (Figure 4b). Thus, this clever computational strategy let us speculate that “RVVPI” and “RIAPA” could prevent the conformational switch of the Aβ_42_ monomer towards aggregation-prone structures [234].

The same in silico protocol to find inhibitors of Aβ_42_ self-association was tested starting from the β-breaker peptide “LPFFD” and the structure of Aβ_42_ in the aggregated fibrillar state (Figure 4b) [235]. “LPFFD” is a known inhibitor of Aβ aggregation that can dissociate fibrils in vitro but also decrease fibrillogenesis and Aβ deposition in models of rat brains. A virtual library of 867 pentapeptides was assembled by incorporating mutations in the starting β-breaker peptide. Virtual screening by docking was first performed using as a target the structure of Aβ_42_ in the fibrillar form (PDB ID: 2BEG [239] Figure 4b), and, afterwards, MM-PBSA analyses and MD simulations were carried out. This approach selected the “PPFFE” peptide as the most promising β-breaker peptide able to interact with the core region of the Aβ_42_ protofibrils, favoring energy minima characterized by a lower β-sheet content and a decrease in the H-bonds [235].

**Figure 5:**
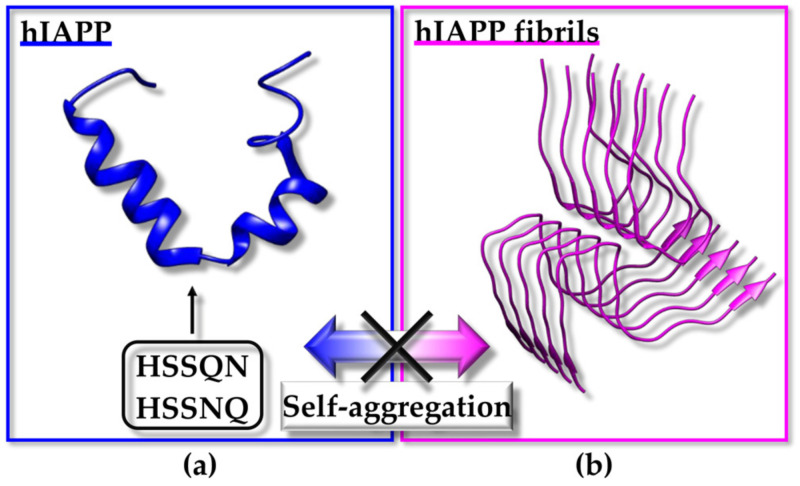
(**a**) NMR structure of hIAPP monomer (blue) calculated in SDS (Sodium Dodecyl Sulphate) micelles, (PDB ID: 2L86 [240], first conformer). The in silico identified HSSQN and HSSNQ peptides could favor the helical state in monomeric hIAPP [236]. (**b**) Cryo-EM structure of hIAPP fibrils (magenta) (PDB ID: 7M61 [241]).

A similar computational strategy was also employed to discover peptide inhibitors of hIAPP (Human islet amyloid polypeptide) self-aggregation that leads to cytotoxic fibers connected to the pathogenicity of type 2 diabetes (Figure 5a,b). Small peptide fragments encompassing the amyloidogenic region of hIAPP work as blockers of self-assembly. In particular, the fragment HSSNN_18–22_ was identified as an amyloidogenic sequence demonstrating elevated antiproliferative properties towards RIN-5F cells. Thus, the “HSSNN” peptide was used as a model to generate a virtual library of mutated peptides. This library was virtually screened against the structure of hIAPP in the monomeric state (PDB entry: 2L86 [240]) (Figure 5a). The subsequent MM-PBSA investigation of the best docking hits pointed out two peptides (HSSQN and HSSNQ) that were able to target monomeric hIAPP with a high affinity. Further MD simulations highlighted that when in a complex with each peptide, monomeric hIAPP underwent the enhanced sampling of helical conformation with a consequent decrease in the aggregation tendency [236].

In conclusion, the in silico approach combining several computational tools (i.e., docking-based VS, MM-PBSA, and MD) represents a suitable instrument to select the most promising peptides, potentially able to block the self-aggregation mechanisms related to AD and type 2 diabetes to be experimentally validated.

## 4. Conclusions

This review can be considered a sort of toolkit for researchers who intend to start a drug discovery study using docking-based virtual screening approaches and peptide libraries to target proteins involved in different diseases.

Most functions within cells are mediated by networks of PPIs, the dysregulation of which is usually related to pathological conditions; thus, there is growing interest in the discovery of compounds able to modulate such interactions positively or negatively. PPIs usually occur through vast surfaces characterized by smoothness and are often unable to be attacked by small molecules. In this framework, peptides, that can also be easily produced by well-established synthetic routes, represent a valid alternative [2,3]. Nevertheless, in comparison to small molecules, peptides can be provided with a higher efficacy and specificity for their protein targets due to their chemical variety as well as conformational properties (i.e., the ability to assume a multiplicity of secondary structure elements along with the capacity to undergo conformational variations when binding to a protein surface) [2,3,242]. During the last few years, tremendous improvements have been made in the medicinal chemistry field related to the production of peptides with better drug-like features, and a variety of synthetic routes to prepare peptides provided with specific secondary structure elements have been set up [35,41]. However, the costs connected to the production of synthetic peptide libraries are relevant, especially if certain non-natural amino acids or cyclic organizations need to be included to increase stability and affinity for the target [242]. It also has to be considered that, when looking for a potential therapeutic peptide acting on a specific protein or PPI, the screening of very large peptide libraries, provided with the proper chemical diversity grade, needs to be carried out to improve the chance of finding a good hit. To perform the initial experimental screenings for evaluating peptide–protein interactions, additional costs, related to the chemicals and materials needed to run the binding assays, must be faced, as well as the costs connected to recombinant protein production (for example, when choosing to perform screening by NMR, ^15^N and/or ^13^C-labeled proteins are often required, but their production is more expensive). Such experimental screenings are also time-consuming.

However, when the target protein or protein–protein interaction has been well characterized from a structural point of view and high-resolution atomic coordinates can be downloaded from PDB, in silico approaches can be very useful in preselecting putative peptide ligands to be submitted to experimental validation, thus reducing the time and costs connected to a drug discovery campaign. Indeed, as described in the previous paragraphs, large libraries of virtual peptides can be generated through a variety of in silico tools, and diverse strategies can be adopted to design the library that best suits the system under study. These libraries can next be implemented in structure-based virtual screening [81,110,112,186,243]. To predict binding poses, peptides can be docked inside the protein binding pockets extracted directly from the complex structure, if available, or alternatively, diverse bioinformatic tools can be implemented to evidence pockets and grooves and establish a putative binding site on the protein surface [151]; even blind docking can be performed, but, in this case, less accurate results might be obtained and longer computational times, connected to the exploration of the whole protein surface, are required [244]. Another aspect to be considered is that, when performing docking-based virtual screening, flexibility is important, and most docking tools allow for a certain treatment of flexibility not only concerning the ligand but also the protein receptor [76,80,82,245]. In the end, docking scores can be employed to preselect a certain number of compounds, but, as selection criteria may not be straightforward enough to be established [246], before starting experimental validations, it can be helpful to further perform MD simulations to obtain some insights into the stability of the predicted docking poses [92,184,214].

All these topics and the major drawbacks have been treated within this review simply to allow the less experienced researchers to gain inspiration for their studies.

Through this review, we describe as well diverse examples of virtual screening approaches to identify peptides targeting a variety of proteins and PPIs involved in cancer, Alzheimer’s disease, diabetes, and COVID-19 [153,187,192,234].

By reviewing the literature, we noticed that many putative peptide ligands have been identified in silico, but often the proper experimental validation of computational results is lacking, and in the end, it is important to consider that a large slice of virtually identified hits might not be able to reach the desired biological effects [81,247]. As much research effort is being spent in the field, and rapid improvements are being achieved, an even larger increase in the success rate of SBVS must be expected in the near future. Meanwhile, it could be interesting to create databases of in silico discovered bioactive compounds and validate those without experimentally proved functions. These data could open additional avenues for generating therapeutic agents and/or setting up novel therapeutic routes.

## Figures and Tables

**Table 1:** ACPs mentioned within this paragraph with the respective protein targets and the computational tools implemented for their identification. “Y” or “N” in the column “Exp.” indicates if experimental validation has been or has not been achieved, respectively. References associated with peptide identification are also reported in the last column.

ACP	Protein Target	Computational Tools	Exp.	Ref.
LYSPH PSYLNTPLL	MPO (PDB ID: 6WYD [136]); XO (PDB ID: 3NVY [137]); Keap1 (PDB ID: 2FLU [138]); p47phox (PDB ID: 1WLP [139])	PlantPepDB [134]; AntiCP 2.0 [135]; PEP-FOLD3 [140]; Autodock Vina [72]/Webina 1.0.3 [141]; HPEPDOCK [71]; MLCPP [142]; B3Pred [143]; PlifePred [144]; BIOPEP-UWM [145]; BUDE Alanine [146]; GROMACS 2020 [87]	N	[133]
LARLLT (D4)	Extracellular domain of EGFR (PDB ID: 1NQL [147])	PSCAN 2.2.2 Autodock 3 [148]	Y	[149]
Ebselen-WE	Kinase domain of EGFR (PDB ID: 3W2S [150])	CASTp [151]; Chemsketch [152]; Autodock Vina [72]	N	[153]
Cyclo- (EIDTVLTPTGWVAKRYS)	CTLA-4 (PDB ID: 1I85 [154])	KFC [155]; Phyre2 [156]; FlexPepDock [157]; PyRosetta [158]; NAMD 2.13 [159]; MM-GBSA calculations [160]; BFEE in VMD [161]	Y	[162]

## Data Availability

No new data were created within this review paper.
